# Trajectories of pharmacological therapies for treatment-resistant depression: a longitudinal study

**DOI:** 10.1186/s12888-025-06518-8

**Published:** 2025-03-10

**Authors:** Julia R. DiBello, Xiaomo Xiong, Xinyue Liu, Wenjun Zhong, Aristide Merola, Minghui Li, Z. Kevin Lu

**Affiliations:** 1 Merck & Co., Inc, Rahway, NJ USA; 2 University of South Carolina College of Pharmacy, 715 Sumter Street, Columbia, SC 29208 USA; 3 James L. Winkle College of Pharmacy, University of Cincinnati Academic Health Center, 3255 Eden Avenue, Cincinnati, OH 45267 USA; 4 University of Tennessee Health Science Center, 881 Madison Avenue, Memphis, TN 38163 USA

**Keywords:** Major depression disorder, Treatment-resistant depression, Antidepressant, Longitudinal study

## Abstract

**Background:**

Treatment-resistant depression (TRD) in major depressive disorder (MDD) is defined as the failure of two or more antidepressants. Few studies have characterized the natural history and treatment patterns of these patients. This study aims to identify the natural history of disease and treatment trajectories for patients with TRD.

**Methods:**

A retrospective longitudinal study used claims data linked to electronic health records (EHRs) from January 1, 2017, to October 31, 2021. Inclusion criteria were age ≥ 18 years, ≥ 1 MDD diagnosis, no antidepressant use at baseline, and an index date within 90 days of MDD diagnosis. Exclusions included psychiatric disorders other than MDD. TRD patients were defined as receiving third-line antidepressant treatment within two years of first-line initiation. Second- and third-line antidepressant treatment was defined as a switch to or addition of a different antidepressant with an adequate dose/duration or initiation of an augmentation treatment.

**Results:**

Out of 301,821 individuals with MDD using antidepressants or augmentation medications during the study, 2,409 incident TRD patients were selected out of 50,374 meeting the criteria. The median time to TRD (time from first to third line index date) was 11.5 months, and the TRD episode duration was 10.8 months. Initial treatment was predominantly antidepressant monotherapy, declining from 91.0% in the first line to 39.4% in the third line. Combination therapy including antidepressants and augmentation medications increased over lines, reaching 55.6% in the third line. During the TRD episode, SSRIs were the most prescribed antidepressants with the longest duration of use. Cognitive-behavioral therapy was used by 53.5% of TRD patients, while other nonpharmacological therapies were rarely used. Treatment trajectories varied by age, sex, and anxiety.

**Conclusions:**

This study identified contemporary treatment patterns in TRD patients, with combination therapy and augmentation medications increasingly used, highlighting the need for precision treatment based on individual trajectories.

**Supplementary Information:**

The online version contains supplementary material available at 10.1186/s12888-025-06518-8.

## Background

Approximately 6.7% of the U.S. population experiences major depressive disorders (MDD), and a significant portion receives pharmacological treatment, which is referred to as pharmaceutically treated depression (PTD) [1, 2]. Within this group, a notable percentage may progress to treatment-resistant depression (TRD) due to failure of response to treatment, low response, or low remission rates [2]. TRD is commonly defined as a lack of response following at least two antidepressant treatments with adequate dose and treatment duration. This condition may impact as many as 30% of MDD patients [2].

Pharmacological treatments during the TRD course can vary significantly across different patient groups and symptom domains [3, 4]. However, few studies have evaluated TRD trajectories or treatment variances across patient groups. TRD typically develops over one to two years and may persist for life [5]. However, most published studies have either had a short follow-up period or were cross-sectional, which did not allow for an examination of the natural history of TRD [5–21].

Moreover, databases used in the literature are mainly claims data from insurers [5–21] These data are generally limited because claims reflect only the diagnoses and services that were submitted for reimbursement purposes [5–21]. Very few studies have used linked data of both claims and electronic health records (EHRs), leading to incomplete information on TRD. Additionally, many published studies used data from before 2017 [5–13]. With the recent FDA approval of the TRD medication in March, 2019, SPRAVATO^®^ (esketamine), it is important to evaluate the effect of new treatment options on treatment trajectories among TRD patients [22–24].

Therefore, using a large nationwide dataset with claims linked with EHR, this research aimed to address critical gaps in the existing literature. By analyzing TRD treatment patterns and identifying variations across patient subgroups, we aimed to provide a comprehensive, descriptive overview of the natural history and treatment trajectories of TRD patients, which will further establish a foundation for future hypothesis-driven investigations into TRD care.

## Methods

### Study design

A retrospective longitudinal study was conducted using the Merative (formerly IBM Watson Health) MarketScan Explorys Claims-EHR Data Set from January 1, 2017, to October 31, 2021 [25]. The MarketScan Explorys Claims-EHR Data Set links claims from the MarketScan Database to corresponding EHRs for the same individuals, with de-identified data.

**Fig. 1:**
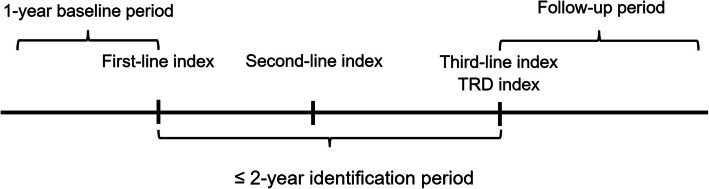
Study design. Note: TRD: Treatment-resistant depression

This study had three periods, including baseline, identification, and follow-up periods. The baseline period was the 1-year period before the index date of first-line treatment. The identification period was defined as ≤ 2-year period after the index date of the first-line treatment [13, 15, 19, 20]. The follow-up period was after the index date of TRD (Fig. 1).

Pharmacological treatments were categorized into treatment lines based on the discontinuation and alteration of antidepressants. The first-line treatment was the initial antidepressant prescription with an adequate dose and duration. Subsequent lines (second and third) were defined as switching to or adding a different antidepressant with adequate dose/duration or initiating augmentation treatment. Details of the antidepressant and augmentation medications are shown in eTable 1 and eTable 2 in Supplementary Materials.

Adequate dose/duration followed American Psychiatric Association guidelines [26] Specifically, all treatment lines were required to have an adequate dose (eTable 1 in Supplementary Materials) and an adequate duration of ≥ 28 days (4 weeks) of supply with gaps of ≤ 14 days (2 weeks). If patients did not achieve an adequate dose/duration on a particular line, they were not considered to have advanced to that line of therapy.

This study received approval from the University of South Carolina institutional review board (IRB), and written consent was waived as all data utilized were de-identified.

### Study population

The study population consisted of a cohort with incident TRD, defined as PTD patients not responding to first- and second-line treatments and progressing to the third-line treatment. Inclusion criteria for the incident TRD cohort included: (1) age ≥ 18 years, (2) presence of ≥ 1 diagnosis of MDD, (3) no prior antidepressant use during baseline, (4) the index date of first-line within 90 days of an MDD diagnosis, and (5) continuous enrollment throughout baseline and identification periods. To ensure the inclusion of incident cases of TRD, patients with no recorded antidepressant use during the 1-year baseline period were selected. This criterion allowed us to focus on individuals initiating pharmacological treatment for MDD within the study period, thereby standardizing the starting point for evaluating treatment trajectories [27–29].

Exclusions included psychiatric disorders (psychosis, schizophrenia, bipolar disorder, and dementia, and other depressive disorders) or mood disorders (obsessive compulsive disorder, adjustment disorder, substance induced mood disorder, and mood disorder due to general illness) other than MDD during baseline. Individuals diagnosed with excluded psychiatric or mood disorders during follow-up were censored at diagnosis time.

### Measurement

Baseline characteristics included age, sex, MDD diagnosis (no MDD, single episode, and recurrent episode), physical (hypertension, diabetes, chronic obstructive pulmonary disease [COPD], and obesity) and mental (anxiety, sleep disorder, and substance use disorder, and suicidality) comorbidities, and Charlson Comorbidity Index (CCI). All diagnoses were identified using the International Classification of Diseases, 10th Revision, Clinical Modification (ICD-10-CM) codes based on the Chronic Conditions Warehouse (CCW) Algorithm. The single and recurrent episodes of MDD were identified using ICD-10-CM codes, with single episodes classified under F32.x and recurrent episodes classified under F33.x. The specific ICD-10-CM codes for MDD diagnoses and comorbidities are detailed in eTable 3.

Outcomes included the natural history of disease (time to TRD, duration of MDD and TRD episode, duration of treatment, remission rate, and relapse rate) and treatment trajectories. Time to TRD denoted the development of TRD, which was the period from the index date of first-line treatment to the index date of TRD. The duration of depression episode includes the duration of the MDD episode and the duration of the TRD episode. Specifically, the start of the MDD episode was the initial diagnosis of MDD or the first prescription of antidepressants or augmentation therapy, while the end of the MDD episode was the last MDD diagnosis or prescription of antidepressants or augmentation therapy in the study period. The duration of the TRD episode began with the TRD index date and with the same end date as the MDD episode. Remission was defined as a period of at least 180 days without the use of antidepressant or augmentation therapy after completing third-line antidepressant treatment. Relapse was defined as a hospitalization with a primary diagnosis of MDD or suicidality after the TRD index date. These definitions for remission and relapse are not validated and were considered exploratory within the context of the study.

Treatment trajectories were based on the chosen regimen for each treatment line, categorized as monotherapy (antidepressant or augmentation) and combination therapy (two or more antidepressants or antidepressants with augmentation). Characteristics of these treatment trajectories included the frequency of use and the duration (total days supplied). Additionally, the use of non-pharmacological treatments included behavioral therapies (cognitive behavioral therapy [CBT]) and procedures (electroconvulsive therapy [ECT], repetitive transcranial magnetic stimulation [rTMS], and Vagus nerve stimulation [VNS]).

### Statistical analysis

Descriptive analysis was used for baseline characteristics, natural history of disease, and treatment trajectories. Descriptive statistics included mean, standard deviation, median, Q25, and Q75 for continuous variables, and percentages for categorical variables. Sankey plots illustrated treatment trajectories for TRD patients, showing the choice of treatment across first-, second-, and third-line treatments. Differences in treatment trajectories by patient characteristics (age, sex, and presence of anxiety) were also examined using Sankey plots. All analyses were conducted using SAS version 9.4 (SAS Institute Inc).

### Subgroup analysis

To explore potential variations within younger adults, we conducted a subgroup analysis by further stratifying the 18–35 age group into two narrower age categories: 18–25 years and 25–35 years. Baseline characteristics and treatment characteristics were compared between these subgroups.

In addition, treatment trajectories were analyzed among TRD patients by age, sex, and anxiety status to identify potential disparities. Superficially, patients were stratified into two age groups (18–35 years and ≥ 35 years), by sex (male and female), and by anxiety status (with and without anxiety). First-, second-, and third-line treatments were identified and categorized by drug classes, presented by Sankey plots.

### Sensitivity analysis

We conducted sensitivity analyses to test the robustness of the results: (1) Not excluding patients with psychiatric disorders other than MDD; (2) not excluding patients with mood disorders other than MDD; (3) changing the definition of the duration of supply to 3 weeks; (4) changing the definition to 6 weeks; (5) requiring that patients have > 1 antidepressant prescription to be considered to have an adequate duration of use; and (6) including patients with a first-line index date within any duration of MDD diagnosis.

## Results

### Baseline characteristics

**Fig. 2:**
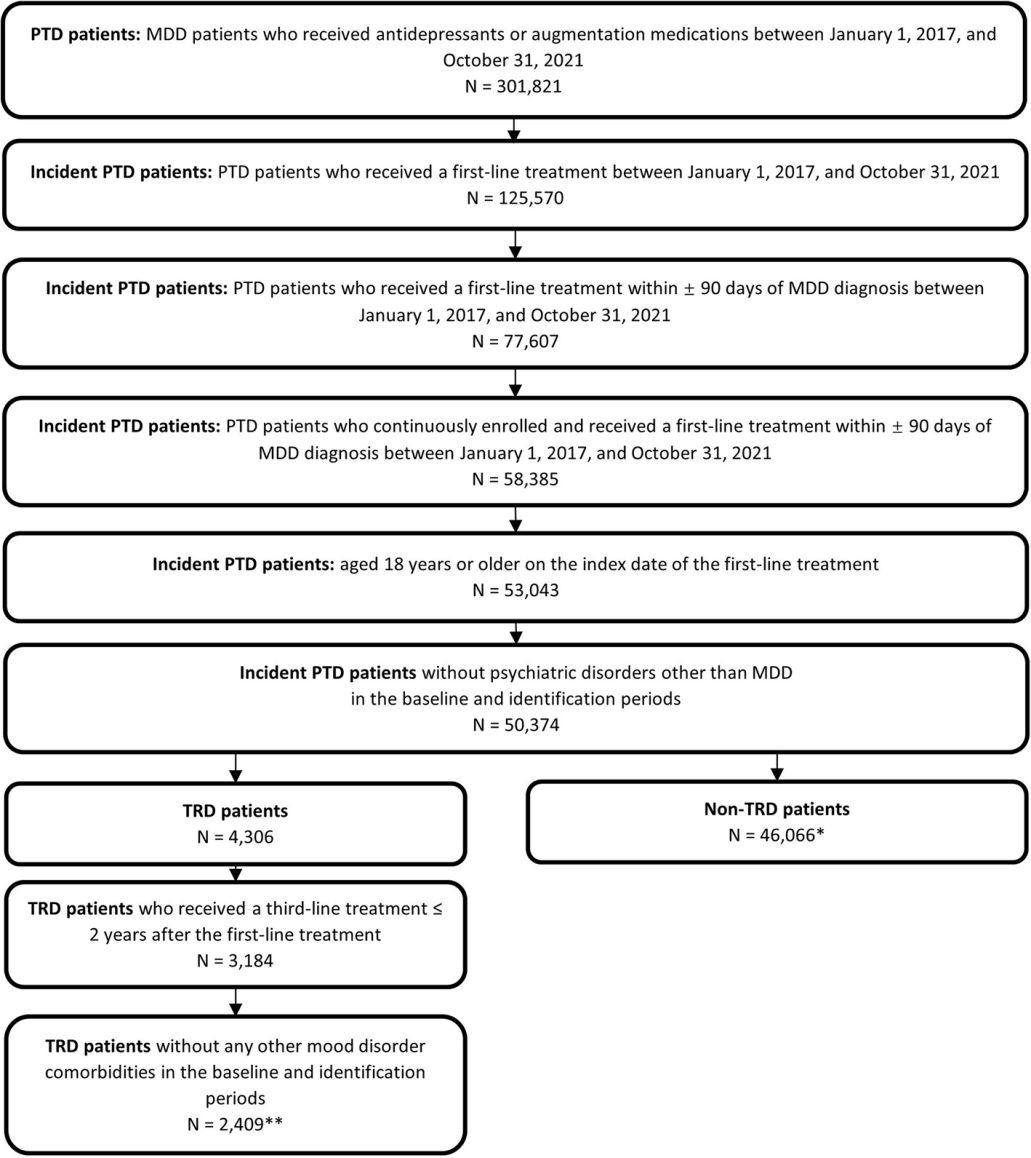
Flowchart on study sample selection. Note: *Two patients who used TRD-approved medication in the first two lines of treatment were excluded; **Out of patients without psychiatric or mood disorders other than MDD MDD: Major Depressive Disorder; PTD: Pharmaceutically treated depression; TRD: Treatment-resistant depression

Out of 301,821 PTD individuals using antidepressants/augmentation, 125,570 were incident PTD patients receiving first-line medication. Among these, 77,607 initiated first-line treatment within 90 days of MDD diagnosis. After excluding patients with psychiatric disorders other than MDD, we included 50,374 incident PTD patients who were continuously enrolled and were ≥ 18 years old on the index date of first-line treatment. A total of 4,306 individuals (8.5%) received third-line treatment and were defined as TRD patients. After further exclusions, 2,409 incident TRD patients (4.8%) were included (Fig. 2).

Table 1 shows the baseline characteristics of the 2,409 incident TRD patients. The mean age was 38.3 (± 15.1) years, with 46.1% aged between 18 and 35 years. Females made up the majority (70.6%) of TRD patients. Of the entire TRD sample, 41.4% had a single episode of MDD, while 32.8% had recurrent episodes during the baseline period. The most common comorbidity was anxiety (47.6%), and 3.3% experienced suicidal events (including suicidal ideation).

**Table 1:** Baseline characteristics of TRD patients (overall, *N* = 2,409)

Variables	*N*	%
**Age**		
≥ 18 and < 35	1,110	46.1
≥ 35 and < 45	506	21.0
≥ 45 and < 55	402	16.7
≥ 55 and < 65	274	11.4
≥ 65	117	4.9
**Sex**		
Male	709	29.4
Female	1,700	70.6
**MDD diagnosis during baseline**		
No MDD during baseline	623	25.9
Single episode	997	41.4
Recurrent episode	789	32.8
**Comorbidities**		
Cancer	33	1.4
Hypertension	441	18.3
Diabetes	173	7.2
Type 1	21	0.9
Type 2	152	6.3
Others^a^	0	0.0
COPD	101	4.2
Obesity^b^	443	18.4
Anxiety	1,146	47.6
Sleep disorder	456	18.9
Substance use disorder	183	7.6
Suicidality	79	3.3
**CCI**		
0	1,836	76.2
1	387	16.1
2	125	5.2
≥ 3	61	2.5

*N *Number, *SD* Standard deviation, *MDD* Major depression disorder, *COPD* Chronic obstructive pulmonary disease, *CCI* Charlson comorbidity index

^a^Others included diabetes mellitus due to underlying condition, drug or chemical-induced diabetes mellitus, and other specified diabetes mellitus

^b^Obesity was identified based on ICD-10 codes

### Natural history of disease

Table 2 demonstrates time to event, treatment duration, episode duration, remission, and relapse for TRD patients. The median time to TRD was 11.5 months (IQR: 7.0, 17.0). The median duration of the MDD episode in TRD patients was 24.6 months (IQR: 16.3, 35.2), with the duration of the TRD episode being 10.8 months (IQR: 4.3, 22.7). This duration meets the Diagnostic and Statistical Manual of Mental Disorders (DSM)−5 criteria for chronic depression, defined as depressive symptoms persisting for two or more years [30]. The duration of each treatment line varied, with a median of 3.7 months (IQR: 2.0, 7.4) for the first-line to the second-line treatment, 4.7 months (IQR: 2.5, 9.2) for the second-line to the third-line treatment, and 10.8 months (IQR: 4.3, 22.7) for the third-line to the end of MDD episode. The median follow-up time after the MDD episode ended until the last enrollment date was 0.6 months (IQR: 0.3, 0.9). Among TRD patients, 29.8% (718/2,409) achieved remission, and 6.2% (150/2,409) experienced relapse.

**Table 2:** Treatment characteristics (time to event, duration of treatment, duration of episode, remission, and relapse) of TRD patients (overall, *N* = 2,409)

**Outcomes**	**Median**	**Q25, Q75**	**Mean**	**SD**
**Time to event**				
Time to TRD, months	11.5	7.0, 17.0	12.2	6.1
**Duration of episode**				
Duration of MDD episode, months	24.6	16.3, 35.2	26.7	13.3
Duration of TRD episode, months	10.8	4.3, 22.7	14.5	12.5
**Duration of treatments**				
Start of the MDD episode to first-line, months	0.0	0.0, 0.7	0.4	0.8
First-line to second-line, months	3.7	2.0, 7.4	5.4	4.5
Second-line to third-line, months	4.7	2.5, 9.2	6.4	5.0
TRD to the end of MDD episode, months	10.8	4.3, 22.7	14.5	12.5
Follow-up after the end of MDD episode, months	0.6	0.3, 0.9	1.9	4.8
**Remission/Relapse (Yes/No)**^**a**^				
Remission (N of individuals (%))	718 (29.8%)			
Relapse (N of individuals (%))	150 (6.2%)			

Start of the MDD episode: first MDD diagnosis or first prescription of antidepressants or augmentation treatments, whichever came first. End of the MDD episode: last MDD diagnosis or last prescription of antidepressants or augmentation treatments, whichever came last

Duration of MDD episode: months between MDD episode start and end (either the date of the last MDD diagnosis or date of the end of the drug supply, whichever is later.)

Duration of TRD episode: months between TRD index date and the end of MDD episode (either the date of the last MDD diagnosis or date of the end of the drug supply, whichever is later.)

Remission in TRD patients: ≥ 180 days gap in antidepressant or augmentation therapy following the end of third-line antidepressant or escalating treatment

Relapse in TRD patients: Hospitalization with a primary diagnosis of MDD or Suicidality after TRD or escalating therapy index date

Remission and relapse definitions are exploratory and have not been validated in the data source

*TRD *Treatment-resistant depression, *MDD* Major depression disorder, *N* Number, *SD* Standard deviation, *Q25* Quantile 25, *Q75* Quantile 75

^a^Presented in number and percentage

### Treatment trajectories

**Fig. 3:**
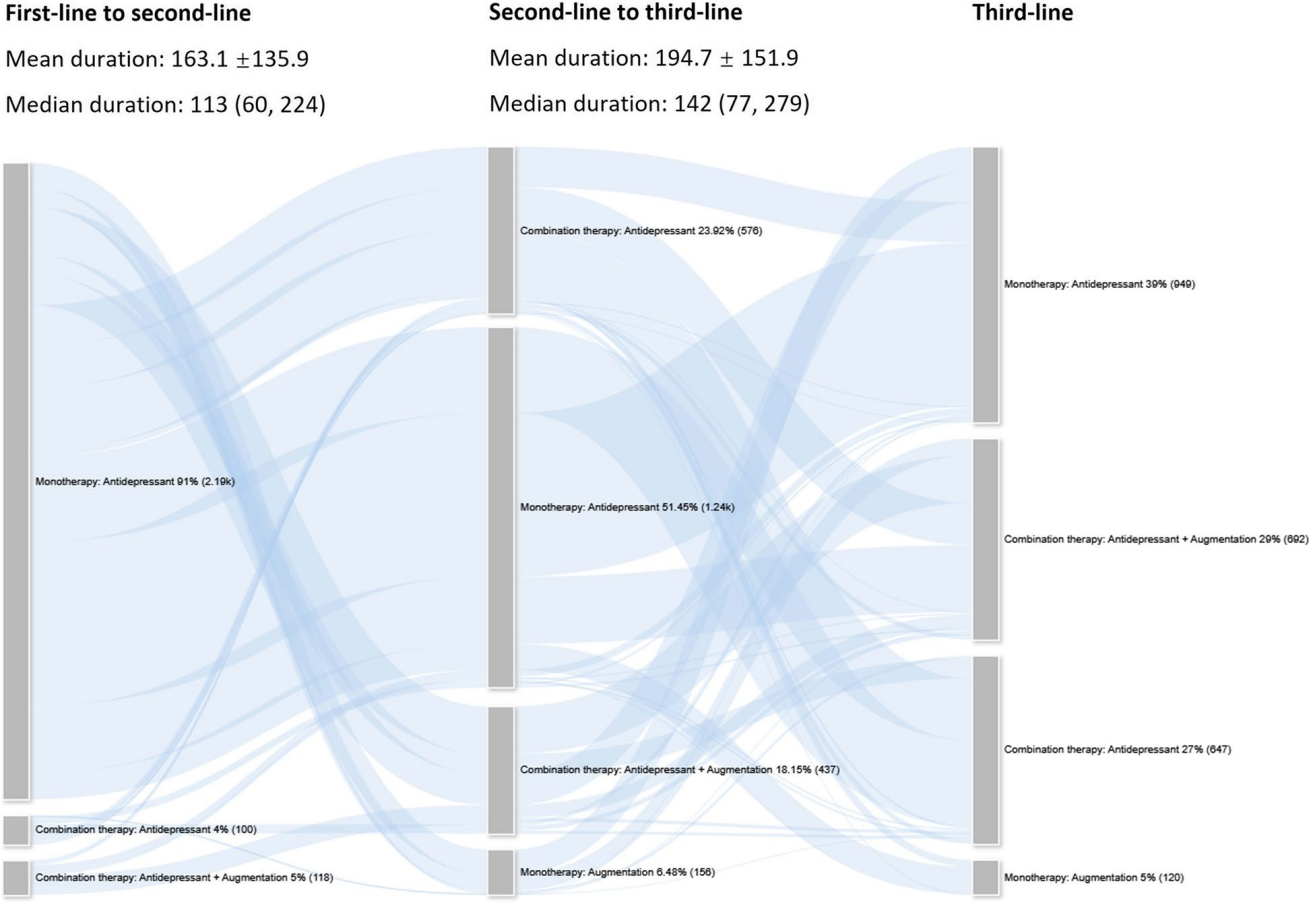
Sankey plot for TRD patients based on monotherapy and combination therapy (*N* = 2,409)

Figure 3 summarizes the treatment trajectories of TRD patients, including monotherapy, combination therapy, and augmentation treatments. Antidepressant monotherapy was the most used treatment across all lines, but its proportion decreased over time: 91.0% (2,191/2,409) in first-line, 51.4% (1,239/2,409) in second-line, and 39.4% (949/2,409) in third-line. Furthermore, during first-line treatment, combination therapy was used by a total of 9.0% (218/2,409) of patients. Specifically, 4.2% (100/2,409) received a combination of antidepressants, while 4.8% (118/2,409) received antidepressants plus augmentation medications. In second-line treatment, there was a greater use of combination treatments (1014/2,409, 42.1%). Moreover, the use of augmentation also increased, including 6.5% (156/2,409) of patients using augmentation monotherapy and 18.2% (437/2,409) of patients using antidepressants plus augmentation medications. In third-line treatment, the proportion of use of combination treatments increased further to 55.6% (1,340/2,409). Specifically, the use of the combination of antidepressants increased from 23.9% (577/2,409) in second-line to 26.9% (648/2,409) in third-line treatment, and antidepressants plus augmentation increased from 18.2% (437/2,409) to 28.7% (692/2,409). Specific trajectories of treatments used in each line are shown in eFigure 1 in Supplementary Materials.

During the MDD episode, considering each medication, SSRIs were the most frequently used antidepressant medication (90.5%), followed by NDRIs (56.7%) and SNRIs (52.3%) (shown in eTable 6 in Supplementary Materials). SSRIs also had the longest median duration of use (8.4 months, IQR: 3.9, 14.1). From the TRD index date to the end of the MDD episode, medication use rates were similar. SSRIs were the most used antidepressants (60.5%), followed by NDRIs (36.8%) and SNRIs (36.3%) (shown in eTable 7 in Supplementary Materials). Moreover, TRD-approved medications, including esketamine and olanzapine combined with fluoxetine, were used by 7.3% in the third-line treatment.

In terms of augmentation treatments, antipsychotics (28.0%) and anxiolytics (27.1%) were the most used (shown in eTable 6 in Supplementary Materials). Both antipsychotics (3.4 months, IQR: 1.7, 7.3) and anxiolytics (3.0 months, IQR: 1.0, 6.6) had a relatively shorter duration of use than other augmentation treatments. On the other hand, although thyroid hormone was not used frequently (0.9%), it had the longest median duration of use (4.7 months, IQR: 1.1, 9.4). From the TRD index date to the end of the MDD episode (eTable 7 in Supplementary Materials), the same pattern persisted, with antipsychotics and anxiolytics being most commonly used but for shorter durations, while thyroid hormone, though infrequent, had a longer duration.

Regarding nonpharmacological treatments, more than half of TRD patients (53.5%) used CBT in their overall treatment, while procedures including ECT, rTMS, and VNS were used by around 1% or fewer of TRD patients (eTable 6 in Supplementary Materials). Moreover, during the period from the TRD index date until the end of the MDD episode, 33.5% of the TRD patients used CBT, while only 0.3%, 0.1%, and 0.5% of the TRD patients used ECT, VNS, and rTMS, respectively (eTable 7 in Supplementary Materials).

### Subgroup analyses

When comparing the baseline characteristics (eTable 4), patients aged 18–25 years were more likely to have comorbid anxiety (*p* = 0.016) and suicidality (*p* = 0.018), while those aged 25–35 years exhibited higher rates of hypertension (*p* < 0.001), obesity (*p* = 0.002), and substance use disorder (0.014). However, no significant differences were observed in treatment characteristics (eTable 5). Moreover, remission and relapse rates were not significantly different between the two groups.

Divergent treatment trajectories were found among TRD patients by age (18 to 35 years and 35 years or older, eFigures 2 and 3 in Supplementary Materials), sex (male and female, eFigures 4 and 5 in Supplementary Materials), and the presence of anxiety (with and without anxiety, eFigures 6 and 7 in Supplementary Materials).

In first-line treatment, TRD patients aged 18–35 primarily used monotherapy of SSRIs (66.5%), NDRIs (11.5%), and SNRIs (10.4%). Conversely, in TRD patients aged 35 or older, SSRIs usage was lower (53.3%), but NDRIs (13.7%) and SNRIs (14.4%) were more prevalent. For second-line treatment, the younger group often used SSRIs (32.5%), SSRIs with augmentation (10.5%), and SNRIs (10.1%). In contrast, patients aged 35 or older used SSRIs (25.4%), SSRIs combined with NDRIs (11.5%), and SNRIs (10.2%).

For second-line treatment, males often chose SSRIs (29.3%), SSRIs with augmentation (9.9%), and SSRIs combined with NDRIs (9.5%). Females preferred SSRIs (28.1%), SSRIs with NDRIs (11.2%), and SNRIs (10.7%).

In third-line treatment, TRD patients without anxiety mainly used SSRIs (17.9%), followed by SSRIs combined with NDRIs (10.1%) and SNRIs (9.8%). TRD patients with anxiety, on the other hand, utilized SSRIs (18.6%), SSRIs added on augmentation treatment (10.8%), and SSRIs combined with NDRIs (10.0%) as their top three treatments.

### Sensitivity analysis

Results of each sensitivity analysis are shown in supplementary tables eTables 8–13 in Supplementary Materials.

## Discussion

This retrospective, longitudinal study described the natural history and treatment trajectories of MDD patients developing TRD. Antidepressant monotherapy was the predominant initial treatment for patients, declining from 91.0% in the first line to 39.4% in the third-line. Combination therapy and augmentation increased, especially in the second and third lines, reaching 55.6% in the third-line. During the TRD episode, SSRIs were the most prescribed antidepressants with the longest duration of use. Our results are consistent with previous studies. In a study by Lundberg et al. from Sweden, in which a population-based observational study was conducted using a regional registry cohort dataset between 2010 and 2018 [31], the most common comorbidity in TRD patients was anxiety, which is consistent with our results [31]. Similarly, this study found that SSRIs were the most frequently used medications across the first three lines of treatments in TRD patients, and that augmentation medications were used increasingly over treatment lines [31]. However, our study found more patients used augmentation mediation compared to the study by Lundberg et al., which might be due to the difference in the clinical practice or marketed drugs for patients with MDD between the United States and Sweden [31] The study by Lundberg et al. also found that psychotherapy was the most frequently used nonpharmacological treatment in TRD patients, and only a few patients used procedures to treat MDD, such as ECT and rTMS [31].

The observed mean duration of depression in our cohort aligns with the DSM-5 definition of chronic depression [30, 32]. This distinction is clinically significant, as chronic depression may require different therapeutic approaches compared to shorter episodes of MDD. The chronic depression in the TRD cohort shows the importance of integrating targeted strategies, such as augmentation therapies or combination treatments, which may be more effective in addressing the persistent and resistant nature of symptoms in this population. In addition, our study found that the median time from the start of MDD to TRD was 11.5 months, which is shorter compared to the 18.1 months found in the study by Lundberg et al. [31]. This might be because we used claims data that may be left-censored for the diagnosis of MDD, resulting in a lagged incident date of MDD episode. In addition, this might be due to the difference in the natural history of our study population, as we used a national population of insured patients, while the study by Lundberg et al. used a regional cohort [31]. However, future studies are still required in the United States, in cohorts with a longer duration of medical history, to validate the period between the start of MDD to the onset of TRD.

In addition, significant contributions to the TRD literature have been made by the European Group for the Study of Resistant Depression (GSRD) in Europe. Our study aligns with the publications by GSRD in several key areas [33–35]. The high prevalence of comorbid anxiety in TRD patients was consistently observed across both U.S. and European populations, showing its critical role in patients with treatment resistance [33–35]. Similarly, the use of augmentation therapies, such as antipsychotics, was a common strategy in both regions, reflecting the need for more intensive treatment approaches as patients progress through multiple lines of therapy [33–35]. Furthermore, the persistent challenge of achieving remission in TRD demonstrates the chronic and refractory nature of this condition across diverse healthcare contexts [33–35]. Despite these similarities, notable differences in treatment trajectories were observed, potentially driven by regional healthcare systems, clinical guidelines, and medication availability. Our study showed a higher reliance on CBT. By contrast, European studies reported lower adoption rates of these interventions, with a greater emphasis on traditional pharmacological strategies and less frequent use of non-pharmacological treatments such as CBT [33–35]. These disparities might be attributed to differences in treatment accessibility, reimbursement policies, and regional practice norms. In addition, our findings align with McEvoy’s perspective on the importance of recognizing and understanding small differences in treatment outcomes [36]. This is crucial in the context of TRD, where subtle variations can significantly impact patient care.

The characteristics and treatment trajectories of TRD patients provide valuable insights, informing future research and clinical practice. The demographic profile, with a mean age of 38.3 years and a majority being female, suggests that healthcare professionals should be particularly focusing on TRD in women and individuals in their late 30s [37, 38]. Alternatively, TRD may be underdiagnosed in certain groups, emphasizing the importance of monitoring all patients for insufficient response to antidepressants. Additionally, with almost half of the patients having co-morbid anxiety, clinicians should carefully assess and manage co-occurring anxiety disorders in TRD patients [39]. The predominance of antidepressant monotherapy, particularly SSRIs, as initial TRD treatments highlights the importance of reassessing their effectiveness. The escalation to combination therapies and augmentation treatments indicates that clinicians recognize the complexity of TRD and are attempting to tailor interventions accordingly. However, the low usage of TRD-approved medications in the third-line treatment (7.3%) may signal a potential gap in guideline adherence or limited access to medications, warranting further investigation and education. Moreover, the stratification of younger patients (18–35 years) into narrower age groups revealed significant differences in baseline characteristics, such as the higher prevalence of anxiety and suicidality in the 18–25 group and higher rates of hypertension, obesity, and substance use disorder in the 25–35 group. Despite these differences, treatment characteristics, including time to TRD, duration of TRD episodes, remission, and relapse rates, were not significantly different between the two groups. These findings underscore the heterogeneity within younger TRD patients and suggest that treatment approaches might need to consider age-related comorbidities to optimize outcomes.

Regarding augmentation treatments, the frequent use of antipsychotics and anxiolytics in TRD, despite their relatively shorter duration of use, underscores the complexity of managing TRD. This complexity arises from the challenges associated with selecting and optimizing augmentation strategies, balancing efficacy with the risk of side effects, such as metabolic disturbances from antipsychotics or dependency potential with anxiolytics [40, 41]. However, the need for frequent augmentation or combination therapies more broadly reflects the multifaceted nature of TRD treatment. The shift from monotherapy to combination therapies, including antidepressants with augmentation agents, highlights the clinical recognition that monotherapy alone often does not suffice for many patients. This observation supports the need for precision treatment approaches tailored to patient-specific characteristics and tolerability. This complexity is further compounded by the side effects of these medications, particularly when combined with antidepressants [42]. These challenges emphasize not only the need for careful monitoring but also new treatment strategies in TRD. The occasional yet prolonged use of thyroid hormone augmentation suggests a potential role for this approach in select cases, meriting additional research to clarify its role in treating TRD [43, 44]. Furthermore, studies suggest that ECT, rTMS, and VNS may offer effective alternative treatments for TRD patients [45]. However, the low utilization rates of these treatments found in this study prompts inquiries into their accessibility and acceptability barriers [45, 46]. Further research is necessary to comprehend the reasons behind the limited use of these procedures.

In addition to augmentation treatments, esketamine, approved by the FDA in 2019 for TRD, was considered a potential influence on treatment trajectories during the study period [24]. However, its use was observed in around 7.3% of third-line treatments, often in combination with other therapies. The low utilization of esketamine could reflect its relatively recent introduction, high cost, or limited availability during the study period [47–49]. These findings suggest that while esketamine holds promise as a novel TRD therapy, its clinical adoption may have been constrained by external factors rather than clinical preference alone [47]. Further research is needed to evaluate its real-world impact on TRD outcomes as more longitudinal data become available.

### Limitations

First, this retrospective study used secondary data and could not assess some characteristics related to TRD patients (e.g., family history, caregiving, and income). The COVID-19 pandemic, particularly in 2020 and 2021, might have impacted outcomes due to elective care cancellations and social distancing, potentially reducing healthcare and medication use. Second, we did not conduct inferential statistical analyses in this study. However, our study establishes a descriptive framework for understanding TRD treatment trajectories. Building on these findings, our future research will employ inferential methods to identify the impact of different treatment trajectories on outcomes, investigate predictors of TRD, and examine disparities in treatment access and quality. Third, only structured data from EHRs was used. The information available in the unstructured EHR data (e.g., patient surveys, habits, problem lists) was not measured. Fourth, the observed prevalence of diabetes in our TRD cohort is lower than expected in the general population. These findings might reflect the limitation of diagnostic coding in claims data. Claims data primarily rely on ICD codes submitted during healthcare encounters. Underdiagnosis of comorbidities such as diabetes among individuals with mental health conditions may be a limitation of claims data, as they are often secondary considerations for billing purposes compared to the primary diagnoses of major depressive disorders. This finding underscores the complexity of comorbidity prevalence in claims-based studies and highlights the need for further investigation into the interplay between depression, diabetes, and associated factors. Similarly, prescription timing in the claims might not align with actual medication use, and real-life medication adherence might also differ from claims medication adherence. Future studies incorporating patient-reported outcomes are needed to better capture treatment trajectories. Fifth, the definitions for remission and relapse were exploratory and not validated, requiring future validation in other datasets or clinical evaluations. Lastly, although esketamine was approved during the study period, its limited adoption in the dataset restricts our ability to evaluate its full impact on treatment patterns and outcomes. Future studies with longer follow-up periods and larger cohorts of esketamine users are necessary to provide a more comprehensive assessment.

## Conclusion

Overall, this study highlights the challenges and complexities of treating TRD patients. The shift from antidepressant monotherapy in first-line to complex combination and augmented therapies in subsequent lines underscores the challenge in effectively treating TRD. Moreover, the differences in treatment trajectories highlight the need for precision treatment plans for TRD patients. Clinicians should consider patient-centered treatment plans that consider the patient’s characteristics, comorbidities, and treatment history to optimize the chance of achieving remission and preventing relapse.

## Supplementary Information

Supplementary Material 1.

## Data Availability

The data used in this study is subject to specific licenses and restrictions by Merative MarketScan Research Databases. Please refer to the Merative guidelines at https://www.merative.com/documents/brief/marketscan-explainer-general.

## Abbreviations

TRD: Treatment-Resistant Depression
MDD: Major Depressive Disorder
EHR: Electronic Health Records
PTD: Pharmaceutically Treated Depression
IRB: Institutional Review Board
COPD: Chronic Obstructive Pulmonary Disease
CCI: Charlson Comorbidity Index
CCW: Chronic Conditions Warehouse
CBT: Cognitive Behavioral Therapy
ECT: Electroconvulsive Therapy
rTMS: Repetitive Transcranial Magnetic Stimulation
VNS: Vagus Nerve Stimulation
IQR: Interquartile Range
SSRIs: Selective Serotonin Reuptake Inhibitors
NDRIs: Norepinephrine-Dopamine Reuptake Inhibitors
SNRIs: Serotonin-Norepinephrine Reuptake Inhibitors
