# Supplementary material for: Trajectories of pharmacological therapies for treatment-resistant depression: a longitudinal study
Source: BMC Psychiatry. 2025 Mar 10;25:215. doi: 10.1186/s12888-025-06518-8 (PMC11892204; doi:10.1186/s12888-025-06518-8)
Supplement: Supplementary file 1 — Supplementary Material 1. [file 12888_2025_6518_MOESM1_ESM.docx]

**Supplementary Material**

**Trajectories of Pharmacological Therapies for Treatment-Resistant Depression: A Longitudinal Study**

Authors: Julia R. DiBello, Ph.D.; Xiaomo Xiong, Ph.D.; Xinyue Liu, Ph.D.; Wenjun Zhong, Ph.D.; Aristide Merola, MD, PhD; Minghui Li, Ph.D.; Z. Kevin Lu, Ph.D., FISPE

[eTable 1. Definition of antidepressant treatment adequate dose](#_Toc184940342)

[eTable 2. List of augmentation treatment](#_Toc184940343)

[eTable 3. ICD-10-CM Codes](#_Toc184940344)

[eTable 4. Comparison of Baseline Characteristics Between Age Groups (18-25 vs. 25-35)](#_Toc184940345)

[eTable 5. Comparison of Treatment Trajectories Between Age Groups (18-25 vs. 25-35)](#_Toc184940346)

[eTable 6. Treatment characteristics (usage, duration, and gap of treatments) since first-line treatment until the end of MDD episode of TRD patients (Overall, N = 2,409)](#_Toc184940347)

[eTable 7. Treatment characteristics (use, duration, and gap of treatments) after the TRD index date until the end of MDD episode of TRD patients (Overall, N = 2,409)](#_Toc184940348)

[eTable 8. Baseline characteristics for the TRD patients in each sensitivity analysis](#_Toc184940349)

[eTable 9. Usage of treatments during the MDD episode for the TRD patients in each sensitivity analysis](#_Toc184940350)

[eTable 10. Mean duration of treatments during the MDD episode for the TRD patients in each sensitivity analysis†](#_Toc184940351)

[eTable 11. Median duration of treatments during the MDD episode for the TRD patients in each sensitivity analysis†](#_Toc184940352)

[eTable 12. Mean gap of treatments during the MDD episode for the TRD patients in each sensitivity analysis‡](#_Toc184940353)

[eTable 13. Median gap of treatments during the MDD episode for the TRD patients in each sensitivity analysis‡](#_Toc184940354)

[eFigure 1. Sankey plot for treatment trajectories of TRD patients (Overall, N = 2,409)](#_Toc184940355)

[eFigure 2. Sankey Plot for treatment trajectories of TRD patients aged between 18 and 35 years (N = 1,110)](#_Toc184940356)

[eFigure 3. Sankey Plot for treatment trajectories of TRD patients aged 35 years or older (N = 1,299)](#_Toc184940357)

[eFigure 4. Sankey Plot for treatment trajectories of male TRD patients (N = 709)](#_Toc184940358)

[eFigure 5. Sankey Plot for treatment trajectories of female TRD patients (N = 1,700)](#_Toc184940359)

[eFigure 6. Sankey Plot for treatment trajectories of TRD patients without anxiety (N = 1,263)](#_Toc184940360)

[eFigure 7. Sankey Plot for treatment trajectories of TRD patients without anxiety (N = 1,146)](#_Toc184940361)

eTable 1. Definition of antidepressant treatment adequate dose

| **Antidepressant medication** | **Minimum daily adequate dose** |
| --- | --- |
| **SSRIs** |  |
| Citalopram | 20 mg |
| Escitalopram | 10 mg |
| Fluvoxamine | 50 mg |
| Fluvoxamine, continuous release | 100 mg |
| Fluoxetine | 20 mg |
| Paroxetine | 20 mg |
| Paroxetine, extended release | 12.5 mg |
| Sertraline | 50 mg |
| Vilazodone | 10 mg |
| **NDRIs** |  |
| Bupropion | 150 mg |
| **SNRIs** |  |
| Desvenlafaxine | 50 mg |
| Duloxetine | 60 mg |
| Levomilnacipran | 20 mg |
| Milnacipran | 12.5 mg |
| Venlafaxine | 37.5 mg |
| **Serotonin modulators** |  |
| Nefazodone | 50 mg |
| Trazodone | 150 mg |
| Vortioxetine | 10 mg |
| **Norepinephrine-serotonin modulators** |  |
| Mirtazapine | 15 mg |
| **Tricyclics and tetracyclics** |  |
| Amitriptyline | 25 mg |
| Amoxapine | 50 mg |
| Clomipramine | 25 mg |
| Doxepin | 25 mg |
| Desipramine | 25 mg |
| Imipramine | 25 mg |
| Maprotiline | 75 mg |
| Nortriptyline | 25 mg |
| Protriptyline | 10 mg |
| Trimipramine | 25 mg |
| **MAOIs** |  |
| Isocarboxazid | 10 mg |
| Moclobemide | 150 mg |
| Opipramol |  |
| Phenelzine | 15 mg |
| Selegiline transdermal | 6 mg |
| Tranylcypromine | 10 mg |
| **FDA-approved TRD medications** |  |
| Esketamine | 28 mg |
| Olanzapine-fluoxetine | 25 mg |

MAOIs: monoamine oxidase inhibitors; NDRIs: norepinephrine-dopamine reuptake inhibitors; SNRIs: serotonin-norepinephrine reuptake inhibitors; SSRIs: selective serotonin reuptake inhibitors

eTable 2. List of augmentation treatment

| **Augmentation treatment** |
| --- |
| **Anticonvulsants** |
| Lamotrigine |
| Valproate |
| **Anxiolytics** |
| Buspirone |
| **Antipsychotics (second-generation or atypical)** |
| Aripiprazole |
| Olanzapine |
| Paliperidone |
| Quetiapine |
| Risperidone |
| Ziprasidone |
| **Lithium** |
| Lithium |
| **Thyroid hormone** |
| Liothyronine |

eTable 3. ICD-10-CM Codes

| Diseases | ICD-10-CM |
| --- | --- |
| MDD | Single episode: F32.0, F32.1, F32.2, F32.3, F32.4, F32.5, F32.9 Recurrent episode: F33.0, F33.1, F33.2, F33.3, F33.40, F33.41, F33.42, F33.9 |
| Psychiatric comorbidities | Psychosis: F23.x, F28.x, F44.89 Schizophrenia: F20.x, F25.x Bipolar disorder: F30.x, F31.x Dementia: F01.x, F02.x, F03.x Other depressive disorders: F32.8x, F33.8x |
| Mood disorders | Obsessive-compulsive disorder: F42.xx Adjustment disorder: F43.xx Substance-induced mood disorder: F10.xx-F16.xx, F18.xx-F19.xx Mood disorder due to general illness: F04.xx-F07.xx, F09.xx |
| Suicidality | T14.91x, X71.x-X83.x, T36.x-T65.x, T71.x, R45.851 |
| Obesity | E66.0, E66.1, E66.2, E66.8, E66.9, Z68.3, Z68.4 |

MDD: Major depression disorder; ICD-10-CM: International Classification of Diseases, 10th Revision, Clinical Modification.

eTable 4. Comparison of Baseline Characteristics Between Younger Age Groups (18-25 vs. 25-35)

| **Variables** | **18 ≤ Age < 25, N = 578** | |  | **25 ≤ Age < 35, N = 532** | | **P-value‡** |
| --- | --- | --- | --- | --- | --- | --- |
|  | **N** | **%** |  | **N** | % |  |
| **Sex** |  |  |  |  |  | 0.379 |
| Male | 178 | 30.8 |  | 151 | 28.4 |  |
| Female | 400 | 69.2 |  | 381 | 71.6 |  |
| **MDD diagnosis during baseline** |  |  |  |  |  | 0.018 |
| No MDD during baseline | 113 | 19.6 |  | 133 | 25.0 |  |
| Single episode | 272 | 47.0 |  | 209 | 39.3 |  |
| Recurrent episode | 193 | 33.4 |  | 190 | 35.7 |  |
| **Comorbidities** |  |  |  |  |  |  |
| Cancer | 0 | 0.0 |  | 0 | 0.0 | N/A |
| Hypertension | 12 | 2.1 |  | 35 | 6.6 | <0.001 |
| Diabetes | 8 | 1.4 |  | 20 | 3.8 | 0.033 |
| Type 1 | 4 | 0.7 |  | 7 | 1.3 |  |
| Type 2 | 4 | 0.7 |  | 13 | 2.4 |  |
| Others* | 0 | 0.0 |  | 0 | 0.0 |  |
| COPD | 19 | 3.3 |  | 10 | 1.9 | 0.142 |
| Obesity† | 69 | 11.9 |  | 99 | 18.6 | 0.002 |
| Anxiety | 334 | 57.8 |  | 269 | 50.6 | 0.016 |
| Sleep disorder | 54 | 9.3 |  | 66 | 12.4 | 0.101 |
| Substance use disorder | 26 | 4.5 |  | 43 | 8.1 | 0.014 |
| Suicidality | 30 | 5.2 |  | 13 | 2.4 | 0.018 |
| **CCI** |  |  |  |  |  | 0.168 |
| 0 | 492 | 85.1 |  | 444 | 83.5 |  |
| 1 | 77 | 13.3 |  | 70 | 13.2 |  |
| 2 | 6 | 1.1 |  | 16 | 3.0 |  |
| ≥ 3 | 3 | 0.5 |  | 2 | 0.3 |  |

Note: N: Number; SD: Standard deviation; MDD: Major depression disorder; COPD: Chronic obstructive pulmonary disease; CCI: Charlson comorbidity index.

*Others included diabetes mellitus due to underlying condition, drug or chemical-induced diabetes mellitus, and other specified diabetes mellitus

†Obesity was identified based on ICD-10 codes.

‡All groups were compared based using chi-sq test.

eTable 5. Comparison of Treatment Characteristics Between Younger Age Groups (18-25 vs. 25-35)

| **Outcomes** | **18 ≤ Age < 25, N = 578** | | | | |  | | **25 ≤ Age < 35, N = 532** | | | | **P-value†** |
| --- | --- | --- | --- | --- | --- | --- | --- | --- | --- | --- | --- | --- |
|  | **Median** | **Q25, Q75** | **Mean** | **SD** |  | | **Median** | | **Q25, Q75** | **Mean** | **SD** |  |
| **Time to event** |  |  |  |  |  | |  | |  |  |  |  |
| Time to TRD, months | 11.1 | 7.2, 16.8 | 12.1 | 6.0 | 0.4 | | 12.0 | | 6.9, 16.9 | 12.3 | 6.2 | 0.60 |
| **Duration of episode** |  |  |  |  |  | |  | |  |  |  |  |
| Duration of MDD episode, months | 23.9 | 16, 33.5 | 25.8 | 12.5 |  | | 23.4 | | 15.7, 34.5 | 26.3 | 13.5 | 0.55 |
| Duration of TRD episode, months | 10.6 | 4.1, 20.5 | 13.7 | 11.7 |  | | 9.8 | | 3.9, 23.1 | 14.0 | 12.5 | 0.70 |
| **Duration of treatments** |  |  |  |  |  | |  | |  |  |  |  |
| Start of the MDD episode to first-line, months | 0.0 | 0, 0.9 | 0.5 | 0.9 |  | | 0.0 | | 0, 0.6 | 0.4 | 0.7 | 0.12 |
| First-line to second-line, months | 3.8 | 2.1, 7.3 | 5.4 | 4.3 |  | | 3.6 | | 2, 7.6 | 5.4 | 4.5 | 0.97 |
| Second-line to third-line, months | 4.6 | 2.5, 8.9 | 6.2 | 4.8 |  | | 5.0 | | 2.5, 9.2 | 6.5 | 4.9 | 0.28 |
| TRD to the end of MDD episode, months | 10.6 | 4.1, 20.5 | 13.7 | 11.7 |  | | 9.8 | | 3.9, 23.1 | 14.0 | 12.5 | 0.70 |
| Follow-up after the end of MDD episode, months | 0.6 | 0.3, 1 | 2.5 | 5.5 |  | | 0.5 | | 0.3, 0.9 | 2.1 | 5.0 | 0.17 |
| **Remission/Relapse (Yes/No)*** |  |  |  |  |  | |  | |  |  |  |  |
| Remission (N of individuals (%)) | 177 (30.6%) |  |  |  |  | | 155 (29.1%) | |  |  |  | 0.59 |
| Relapse (N of individuals (%)) | 49 (8.5%) |  |  |  |  | | 39 (7.3%) | |  |  |  | 0.48 |

Note: TRD: Treatment-resistant depression; MDD: Major depression disorder; N: Number; SD: Standard deviation; Q25: Quantile 25; Q75: Quantile 75.

*Presented in number and percentage.

†Continuous variables were compared using t-test and categorical variables were compared using chi-sq test.

Notes: Start of the MDD episode: first MDD diagnosis or first prescription of antidepressants or augmentation treatments, whichever came first. End of the MDD episode: last MDD diagnosis or last prescription of antidepressants or augmentation treatments, whichever came last.

Duration of MDD episode: months between MDD episode start and end (either the date of the last MDD diagnosis or date of the end of the drug supply, whichever is later.)

Duration of TRD episode: months between TRD index date and the end of MDD episode (either the date of the last MDD diagnosis or date of the end of the drug supply, whichever is later.)

Remission in TRD patients: ≥ 180 days gap in antidepressant or augmentation therapy following the end of third-line antidepressant or escalating treatment.

Relapse in TRD patients: Hospitalization with a primary diagnosis of MDD or Suicidality after TRD or escalating therapy index date.

Remission and relapse definitions are exploratory and have not been validated in the data source.

eTable 6. Treatment characteristics (usage, duration, and gap of treatments) since first-line treatment until the end of MDD episode of TRD patients (Overall, N = 2,409)

| **Outcomes** | **Use** | **%** | **Duration^†^, days** | | | |  | **Gap^‡^, days** | | | |
| --- | --- | --- | --- | --- | --- | --- | --- | --- | --- | --- | --- |
|  |  |  | **Mean** | **SD** | **Median** | **Q25, Q75** |  | **Mean** | **SD** | **Median** | **Q25, Q75** |
| **Antidepressants*** |  |  |  |  |  |  |  |  |  |  |  |
| SSRIs | 2,181 | 90.5 | 291.4 | 197.0 | 257.0 | 120, 429 |  | 116.2 | 145.4 | 53.0 | 7, 171 |
| NDRIs | 1,366 | 56.7 | 214.2 | 187.3 | 148.0 | 60, 327 |  | 54.8 | 96.6 | 11.0 | 0, 63 |
| SNRIs | 1,261 | 52.3 | 219.0 | 190.1 | 150.0 | 60, 330 |  | 57.0 | 99.3 | 13.0 | 0, 65 |
| Serotonin modulators | 832 | 34.5 | 171.0 | 167.2 | 90.0 | 50, 247 |  | 68.7 | 115.0 | 10.0 | 0, 88.5 |
| Norepinephrine-serotonin modulators | 330 | 13.7 | 138.2 | 141.6 | 84.0 | 30, 189 |  | 41.0 | 92.5 | 1.0 | 0, 35 |
| Tricyclics and tetracyclics | 406 | 16.9 | 161.6 | 172.1 | 90.0 | 30, 229 |  | 47.8 | 92.5 | 4.0 | 0, 50 |
| MAOIs | 2 | 0.1 | 65.5 | 14.8 | 65.5 | 55, 76 |  | 4.0 | 5.7 | 4.0 | 0, 8 |
| TRD approved medications^§^ | 176 | 7.3 | 48.0 | 45.3 | 30.0 | 30, 60 |  | 45.1 | 118.9 | 0.0 | 0, 8.5 |
| **Augmentation treatments*** |  |  |  |  |  |  |  |  |  |  |  |
| Anticonvulsants | 235 | 9.8 | 183.2 | 174.9 | 120.0 | 47, 262 |  | 38.1 | 71.8 | 7.0 | 0, 46 |
| Anxiolytics | 652 | 27.1 | 145.0 | 146.7 | 90.0 | 30, 199.5 |  | 58.9 | 104.8 | 9.0 | 0, 71 |
| Antipsychotics | 674 | 28.0 | 160.9 | 152.5 | 102.0 | 51, 223 |  | 51.7 | 92.5 | 6.0 | 0, 62 |
| Lithium | 62 | 2.6 | 140.3 | 138.1 | 65.5 | 30, 234 |  | 47.5 | 99.3 | 4.5 | 0, 42 |
| Thyroid hormone | 21 | 0.9 | 195.1 | 193.3 | 143.0 | 34, 286 |  | 37.6 | 52.4 | 2.0 | 0, 70 |
| **Nonpharmacological Treatments** |  |  |  |  |  |  |  |  |  |  |  |
| **Behavioral therapies** |  |  |  |  |  |  |  |  |  |  |  |
| CBT | 1,290 | 53.5 | - | - | - | - |  | - | - | - | - |
| **Procedures** |  |  |  |  |  |  |  |  |  |  |  |
| ECT | 14 | 0.6 | - | - | - | - |  | - | - | - | - |
| VNS | 2 | 0.1 | - | - | - | - |  | - | - | - | - |
| rTMS | 28 | 1.2 | - | - | - | - |  | - | - | - | - |

SSRIs: Selective serotonin reuptake inhibitors; NDRIs: Norepinephrine and dopamine reuptake inhibitors; SNRIs: Serotonin and norepinephrine reuptake inhibitors; MAOIs: monoamine oxidase inhibitors; TRD: Treatment resistant depression. CBT: Cognitive behavioral therapy; ECT: Electroconvulsive therapy; VNS: Vagus nerve stimulation; rTMS: Repetitive transcranial magnetic stimulation.

*Medications of different types of antidepressants are shown Appendix Table 4. Augmentation treatments are shown in Appendix Table 5.

†Duration: Total number of days supplied.

‡Gap: The date of the last claim + days supplied of the last claim – the date of the first claim – total number of days supplied.

^§^Included esketamine and olanzapine in combination with fluoxetine.

eTable 7. Treatment characteristics (use, duration, and gap of treatments) after the TRD index date until the end of MDD episode of TRD patients (Overall, N = 2,409)

| **Outcomes** | **Use** | **%** | **Duration^†^, days** | | | |  | **Gap^‡^, days** | | | |
| --- | --- | --- | --- | --- | --- | --- | --- | --- | --- | --- | --- |
|  |  |  | **Mean** | **SD** | **Median** | **Q25, Q75** |  | **Mean** | **SD** | **Median** | **Q25, Q75** |
| **Antidepressants*** |  |  |  |  |  |  |  |  |  |  |  |
| SSRIs | 1,457 | 60.5 | 209.0 | 215.3 | 127.0 | 60, 270 |  | 57.4 | 120.1 | 6.0 | 0, 57 |
| NDRIs | 887 | 36.8 | 207.5 | 227.5 | 120.0 | 57, 267 |  | 47.2 | 101.7 | 3.0 | 0, 45 |
| SNRIs | 874 | 36.3 | 184.1 | 211.2 | 90.0 | 39, 247 |  | 38.5 | 96.1 | 2.0 | 0, 30 |
| Serotonin modulators | 527 | 21.9 | 152.9 | 185.8 | 90.0 | 30, 180 |  | 42.9 | 103.0 | 0.0 | 0, 28 |
| Norepinephrine-serotonin modulators | 199 | 8.3 | 148.5 | 191.5 | 82.0 | 30, 178 |  | 36.7 | 98.5 | 0.0 | 0, 25 |
| Tricyclics and tetracyclics | 268 | 11.1 | 126.3 | 151.9 | 87.5 | 30, 144.5 |  | 27.5 | 72.6 | 0.0 | 0, 23 |
| MAOIs | 2 | 0.1 | 65.5 | 14.8 | 65.5 | 55, 76 |  | 4.0 | 5.7 | 4.0 | 0, 8 |
| TRD approved medications^§^ | 175 | 7.3 | 37.2 | 24.3 | 30.0 | 30, 30 |  | 21.6 | 82.0 | 0.0 | 0, 0 |
| **Augmentation treatments*** |  |  |  |  |  |  |  |  |  |  |  |
| Anticonvulsants | 189 | 7.8 | 196.1 | 240.7 | 97.0 | 30, 251 |  | 30.8 | 60.8 | 3.0 | 0, 43 |
| Anxiolytics | 479 | 19.9 | 132.3 | 162.9 | 60.0 | 30, 158 |  | 43.5 | 101.8 | 0.0 | 0, 37 |
| Antipsychotics | 489 | 20.3 | 151.1 | 179.8 | 90.0 | 30, 180 |  | 44.8 | 112.6 | 0.0 | 0, 34 |
| Lithium | 47 | 2.0 | 152.6 | 192.3 | 87.0 | 30, 153 |  | 49.7 | 140.9 | 2.0 | 0, 40 |
| Thyroid hormone | 16 | 0.7 | 221.7 | 187.9 | 191.0 | 82.5, 278.5 |  | 100.7 | 197.2 | 25.5 | 0, 117.5 |
| **Nonpharmacological Treatments** |  |  |  |  |  |  |  |  |  |  |  |
| **Behavioral therapies** |  |  |  |  |  |  |  |  |  |  |  |
| Cognitive behavioral therapy (CBT) | 806 | 33.5 | - | - | - | - |  | - | - | - | - |
| **Procedures** |  |  |  |  |  |  |  |  |  |  |  |
| Electroconvulsive therapy (ECT) | 7 | 0.3 | - | - | - | - |  | - | - | - | - |
| Vagus nerve stimulation (VNS) | 2 | 0.1 | - | - | - | - |  | - | - | - | - |
| Repetitive transcranial magnetic stimulation (rTMS) | 12 | 0.5 | - | - | - | - |  | - | - | - | - |

SSRIs: Selective serotonin reuptake inhibitors; NDRIs: Norepinephrine and dopamine reuptake inhibitors; SNRIs: Serotonin and norepinephrine reuptake inhibitors; MAOIs: monoamine oxidase inhibitors; TRD: Treatment resistant depression. CBT: Cognitive behavioral therapy; ECT: Electroconvulsive therapy; VNS: Vagus nerve stimulation; rTMS: Repetitive transcranial magnetic stimulation.

*Medications of different types of antidepressants are shown Appendix Table 4. Augmentation treatments are shown in Appendix Table 5.

†Duration: Total number of days supplied.

‡Gap: The date of the last claim + days supplied of the last claim – the date of the first claim – total number of days supplied.

^§^Included esketamine and olanzapine in combination with fluoxetine.

eTable 8. Baseline characteristics for the TRD patients in each sensitivity analysis

|  | **Base analysis, N = 2,409,**  **N (%) or mean (SD)** | **Sensitivity analysis 3 N = 2,512,**  **N (%) or mean (SD)** | **Sensitivity analysis 4 N = 3,184,**  **N (%) or mean (SD)** | **Sensitivity analysis 5.1 N = 2,784,**  **N (%) or mean (SD)** | **Sensitivity analysis 5.2 N = 1,217,**  **N (%) or mean (SD)** | **Sensitivity analysis 6 N = 2,474,**  **N (%) or mean (SD)** | **Sensitivity analysis 7 N = 4,101,**  **N (%) or mean (SD)** |
| --- | --- | --- | --- | --- | --- | --- | --- |
| **Age, mean** | 38.3 (15.1) | 38.8 (15.5) | 38.2 (15.0) | 38.4 (15.3) | 40.7 (16.3) | 39.8 (15.4) | 38.8 (14.9) |
| ≥ 18 and < 35 | 1110 (46.1) | 1141 (45.4) | 1474 (46.3) | 1287 (46.2) | 565 (41.3) | 781 (42.1) | 1814 (44.2) |
| ≥ 35 and < 45 | 506 (21.0) | 520 (20.7) | 661 (20.8) | 574 (20.6) | 286 (20.9) | 400 (21.6) | 887 (21.6) |
| ≥ 45 and < 55 | 402 (16.7) | 417 (16.6) | 537 (16.9) | 454 (16.3) | 228 (16.7) | 321 (17.3) | 705 (17.2) |
| ≥ 55 and < 65 | 274 (11.4) | 293 (11.7) | 366 (11.5) | 327 (11.7) | 182 (13.3) | 244 (13.1) | 502 (12.2) |
| ≥ 65 | 117 (4.9) | 141 (5.6) | 146 (4.6) | 142 (5.1) | 108 (7.9) | 110 (5.9) | 193 (4.7) |
| **Gender** |  |  |  |  |  |  |  |
| Male | 709 (29.4) | 742 (29.5) | 983 (30.9) | 827 (29.7) | 408 (29.8) | 551 (29.7) | 1189 (29.0) |
| Female | 1700 (70.6) | 1770 (70.5) | 2201 (69.1) | 1957 (70.3) | 961 (70.2) | 1305 (70.3) | 2912 (71.0) |
| **MDD diagnosis during baseline** |  |  |  |  |  |  |  |
| No MDD diagnosis during baseline | 623 (25.9) | 645 (25.7) | 756 (23.7) | 736 (26.4) | 379 (27.7) | 508 (27.4) | 2225 (54.3) |
| Single episode | 997 (41.4) | 1029 (41.0) | 1295 (40.7) | 1156 (41.5) | 525 (38.3) | 707 (38.1) | 1059 (25.8) |
| Recurrent episode | 789 (32.8) | 838 (33.4) | 1133 (35.6) | 892 (32.0) | 465 (34.0) | 641 (34.5) | 817 (19.9) |
| **Comorbidities** |  |  |  |  |  |  |  |
| Cancer | 33 (1.4) | 37 (1.5) | 51 (1.6) | 39 (1.4) | 31 (2.3) | 21 (1.1) | 62 (1.5) |
| Hypertension | 441 (18.3) | 477 (19.0) | 604 (19.0) | 516 (18.5) | 308 (22.5) | 390 (21.0) | 774 (18.9) |
| Diabetes | 173 (7.2) | 187 (7.4) | 229 (7.2) | 210 (7.5) | 128 (9.4) | 157 (8.5) | 301 (7.3) |
| Type 1 | 21 (0.9) | 21 (0.8) | 29 (0.9) | 27 (1.0) | 13 (1.0) | 21 (1.1) | 39 (1.0) |
| Type 2 | 152 (6.3) | 166 (6.6) | 200 (6.3) | 182 (6.5) | 115 (8.4) | 134 (7.2) | 261 (6.4) |
| Others* | 0 (0) | 0 (0) | 0 (0) | 1 (0) | 0 (0) | 2 (0.1) | 1 (0) |
| COPD | 101 (4.2) | 107 (4.3) | 142 (4.5) | 124 (4.5) | 53 (3.9) | 79 (4.3) | 189 (4.6) |
| Obesity† | 443 (18.4) | 461 (18.4) | 573 (18.0) | 500 (18.0) | 211 (15.4) | 318 (17.1) | 682 (16.6) |
| Anxiety | 1146 (47.6) | 1204 (47.9) | 1693 (53.2) | 1317 (47.3) | 632 (46.2) | 941 (50.7) | 1898 (46.3) |
| Sleep disorder | 456 (18.9) | 475 (18.9) | 650 (20.4) | 519 (18.6) | 212 (15.5) | 365 (19.7) | 726 (17.7) |
| Substance use disorder | 183 (7.6) | 192 (7.6) | 479 (15.0) | 205 (7.4) | 110 (8.0) | 147 (7.9) | 300 (7.3) |
| Suicidality | 79 (3.3) | 85 (3.4) | 182 (5.7) | 91 (3.3) | 43 (3.1) | 68 (3.7) | 113 (2.8) |
| **CCI** |  |  |  |  |  |  |  |
| 0 | 1836 (76.2) | 1892 (75.3) | 2385 (74.9) | 2108 (75.7) | 1046 (76.4) | 1394 (75.1) | 3100 (75.6) |
| 1 | 387 (16.1) | 408 (16.2) | 521 (16.4) | 453 (16.3) | 198 (14.5) | 310 (16.7) | 667 (16.3) |
| 2 | 125 (5.2) | 134 (5.3) | 175 (5.5) | 147 (5.3) | 67 (4.9) | 100 (5.4) | 214 (5.2) |
| ≥ 3 | 61 (2.5) | 78 (3.1) | 103 (3.2) | 76 (2.7) | 58 (4.2) | 52 (2.8) | 120 (2.9) |

N: Number; SD: Standard deviation; MDD: Major depression disorder; COPD: Chronic obstructive pulmonary disease; CCI: Charlson comorbidity index.

Note: *Others included diabetes mellitus due to underlying condition, drug or chemical induced diabetes mellitus, and other specified diabetes mellitus †Obesity was identified Based on ICD-10 codes.

eTable 9. Usage of treatments during the MDD episode for the TRD patients in each sensitivity analysis

|  | **Base analysis, N = 2,409,**  **N (%)** | **Sensitivity analysis 1 N = 2,512,**  **N (%)** | **Sensitivity analysis 2 N = 3,184,**  **N (%)** | **Sensitivity analysis 3 N = 2,784,**  **N (%)** | **Sensitivity analysis 4 N = 1,217,**  **N (%)** | **Sensitivity analysis 5 N = 2,474,**  **N (%)** | **Sensitivity analysis 6 N = 4,101,**  **N (%)** |
| --- | --- | --- | --- | --- | --- | --- | --- |
| **Antidepressants^*^** |  |  |  |  |  |  |  |
| SSRIs | 2181 (90.5) | 2272 (90.4) | 2866 (90.0) | 2525 (90.7) | 1109 (91.1) | 1645 (88.6) | 3706 (90.4) |
| NDRIs | 1366 (56.7) | 1418 (56.4) | 1783 (56.0) | 1585 (56.9) | 737 (60.6) | 1106 (59.6) | 2225 (54.3) |
| SNRIs | 1261 (52.3) | 1316 (52.4) | 1665 (52.3) | 1437 (51.6) | 670 (55.1) | 1007 (54.3) | 2136 (52.1) |
| Serotonin modulators | 832 (34.5) | 876 (34.9) | 1172 (36.8) | 940 (33.8) | 456 (37.5) | 699 (37.7) | 1389 (33.9) |
| Norepinephrine-serotonin modulators | 330 (13.7) | 354 (14.1) | 490 (15.4) | 387 (13.9) | 151 (12.4) | 293 (15.8) | 533 (13) |
| Tricyclics and tetracyclics | 406 (16.9) | 428 (17.0) | 552 (17.3) | 466 (16.7) | 194 (15.9) | 303 (16.3) | 806 (19.7) |
| MAOIs | 2 (0.1) | 2 (0.1) | 6 (0.2) | 2 (0.1) | 4 (0.3) | 6 (0.3) | 6 (0.1) |
| TRD approved medications^§^ | 176 (7.3) | 177 (7.0) | 176 (5.5) | 172 (6.2) | 45 (3.7) | 55 (3.0) | 177 (4.3) |
| **Augmentation treatments^*^** |  |  |  |  |  |  |  |
| Anticonvulsants | 235 (9.8) | 252 (10.0) | 333 (10.5) | 278 (10.0) | 148 (12.2) | 226 (12.2) | 375 (9.1) |
| Anxiolytics | 652 (27.1) | 684 (27.2) | 848 (26.6) | 751 (27.0) | 339 (27.9) | 565 (30.4) | 1161 (28.3) |
| Antipsychotics | 674 (28.0) | 718 (28.6) | 982 (30.8) | 782 (28.1) | 368 (30.2) | 652 (35.1) | 1093 (26.7) |
| Lithium | 62 (2.6) | 76 (3.0) | 86 (2.7) | 70 (2.5) | 33 (2.7) | 61 (3.3) | 94 (2.3) |
| Thyroid hormone | 21 (0.9) | 22 (0.9) | 30 (0.9) | 22 (0.8) | 11 (0.9) | 25 (1.3) | 51 (1.2) |
| **Nonpharmacological Treatments** |  |  |  |  |  |  |  |
| **Behavioral therapies** |  |  |  |  |  |  |  |
| CBT | 1290 (53.5) | 1352 (53.8) | 1894 (59.5) | 1449 (52.0) | 701 (57.6) | 1019 (54.9) | 2062 (50.3) |
| **Procedures** |  |  |  |  |  |  |  |
| ECT | 14 (0.6) | 17 (0.7) | 20 (0.6) | 14 (0.5) | 6 (0.5) | 14 (0.8) | 19 (0.5) |
| VNS | 2 (0.1) | 2 (0.1) | 2 (0.1) | 3 (0.1) | 2 (0.2) | 5 (0.3) | 5 (0.1) |
| rTMS | 28 (1.2) | 30 (1.2) | 41 (1.3) | 29 (1) | 23 (1.9) | 26 (1.4) | 49 (1.2) |

SSRIs: Selective serotonin reuptake inhibitors; NDRIs: Norepinephrine and dopamine reuptake inhibitors; SNRIs: Serotonin and norepinephrine reuptake inhibitors; MAOIs: monoamine oxidase inhibitors; TRD: Treatment resistant depression. CBT: Cognitive behavioral therapy; ECT: Electroconvulsive therapy; VNS: Vagus nerve stimulation; rTMS: Repetitive transcranial magnetic stimulation.

*Medications of different types of antidepressants are shown Appendix Table 4. Augmentation treatments are shown in Appendix Table 5.

^§^Included esketamine and olanzapine in combination with fluoxetine.

eTable 10. Mean duration of treatments during the MDD episode for the TRD patients in each sensitivity analysis†

|  | **Base analysis, N = 2,409, mean (SD)** | **Sensitivity analysis 1 N = 2,512, mean (SD)** | **Sensitivity analysis 2 N = 3,184, mean (SD)** | **Sensitivity analysis 3 N = 2,784, mean (SD)** | **Sensitivity analysis 4 N = 1,217, mean (SD)** | **Sensitivity analysis 5**  **N = 2,474, mean (SD)** | **Sensitivity analysis 6 N = 4,101, mean (SD)** |
| --- | --- | --- | --- | --- | --- | --- | --- |
| **Antidepressants*** |  |  |  |  |  |  |  |
| SSRIs | 291.4 (197.0) | 291.4 (197.7) | 284.1 (196.2) | 280.7 (195.9) | 340.0 (192.6) | 329.2 (196.3) | 292.2 (197.1) |
| NDRIs | 214.2 (187.3) | 214.4 (187.4) | 203.0 (183.1) | 204.8 (186.2) | 252.1 (183.6) | 247.1 (190.8) | 208.2 (184.0) |
| SNRIs | 219.0 (190.1) | 220.7 (191.5) | 217.7 (191.1) | 213.2 (189.6) | 269.1 (193.9) | 260.8 (199.3) | 218.3 (188.3) |
| Serotonin modulators | 171.0 (167.2) | 176.4 (172.0) | 174.1 (169.4) | 169.9 (168.4) | 202.7 (177.2) | 199.0 (187.1) | 171.1 (167.9) |
| Norepinephrine-serotonin modulators | 138.2 (141.6) | 140.4 (142.7) | 138.4 (144.2) | 132.2 (142.3) | 192.5 (167.7) | 171.7 (165.5) | 132.9 (136.4) |
| Tricyclics and tetracyclics | 161.6 (172.1) | 159.7 (169.7) | 156.4 (169.7) | 151.2 (167) | 188.6 (173.1) | 194.2 (180.1) | 165.7 (171.7) |
| MAOIs | 65.5 (14.8) | 65.5 (14.8) | 206.7 (208.5) | 65.5 (14.8) | 110 (97.9) | 132.7 (111.3) | 86 (50.9) |
| TRD approved medications^§^ | 48.0 (45.3) | 47.8 (45.2) | 48 (45.3) | 47.8 (45.6) | 67.3 (71.3) | 54.8 (54.6) | 48 (45.2) |
| **Augmentation treatments*** |  |  |  |  |  |  |  |
| Anticonvulsants | 183.2 (174.9) | 185.5 (180.3) | 185.5 (173.4) | 179.0 (175.0) | 220.0 (178.9) | 202.2 (175.7) | 185.8 (173.7) |
| Anxiolytics | 145.0 (146.7) | 145.2 (146.0) | 142.9 (145.4) | 137.7 (139.8) | 183.6 (150.9) | 167.9 (150.5) | 139.5 (139.6) |
| Antipsychotics | 160.9 (152.5) | 167.6 (159.8) | 162.8 (154.0) | 159.2 (152.6) | 192.1 (157.5) | 182.1 (159.2) | 153.5 (144.7) |
| Lithium | 140.3 (138.1) | 158.8 (160.7) | 133.9 (131.9) | 142.4 (154.6) | 164.8 (131.0) | 168.4 (173.9) | 140.0 (142.8) |
| Thyroid hormone | 195.1 (193.3) | 198.3 (189.2) | 179.7 (171.4) | 190.0 (190.7) | 288.0 (191.1) | 262.8 (185.7) | 234.2 (191.5) |

SSRIs: Selective serotonin reuptake inhibitors; NDRIs: Norepinephrine and dopamine reuptake inhibitors; SNRIs: Serotonin and norepinephrine reuptake inhibitors; MAOIs: monoamine oxidase inhibitors; TRD: Treatment resistant depression. CBT: Cognitive behavioral therapy; ECT: Electroconvulsive therapy; VNS: Vagus nerve stimulation; rTMS: Repetitive transcranial magnetic stimulation.

*Medications of different types of antidepressants are shown Appendix Table 4. Augmentation treatments are shown in Appendix Table 5.

†Duration: Total number of days supplied.

^§^Included esketamine and olanzapine in combination with fluoxetine.

eTable 11. Median duration of treatments during the MDD episode for the TRD patients in each sensitivity analysis†

|  | **Base analysis, N = 2,409, median (IQR)** | **Sensitivity analysis 1 N = 2,512, median (IQR)** | **Sensitivity analysis 2 N = 3,184, median (IQR)** | **Sensitivity analysis 3 N = 2,784, median (IQR)** | **Sensitivity analysis 4 N = 1,217, median (IQR)** | **Sensitivity analysis 5 N = 2,474, median (IQR)** | **Sensitivity analysis 6 N = 4,101, median (IQR)** |
| --- | --- | --- | --- | --- | --- | --- | --- |
| **Antidepressants*** |  |  |  |  |  |  |  |
| SSRIs | 257 (120, 429) | 256 (120, 432.5) | 243 (119, 417) | 239 (117, 416) | 316 (179, 489) | 298 (165, 477) | 260 (120, 436) |
| NDRIs | 148 (60, 326) | 148 (60, 325) | 140 (60, 300) | 140 (59, 304) | 207 (102, 355) | 200.5 (87, 354) | 146 (60, 308) |
| SNRIs | 150 (60, 330) | 150 (60, 334) | 148 (60, 329) | 146 (60, 322) | 211.5 (108, 396) | 198 (90, 388) | 150 (60, 325.5) |
| Serotonin modulators | 90 (50, 247) | 93.5 (52.5, 261) | 100.5 (51, 257.5) | 90 (45, 239.5) | 143.5 (60, 293) | 120 (58, 293) | 90 (50, 244) |
| Norepinephrine-serotonin modulators | 84 (30, 189) | 87 (30, 195) | 85.5 (30, 181) | 64 (30, 180) | 123 (60, 288) | 110 (55, 260) | 83 (30, 185) |
| Tricyclics and tetracyclics | 90 (30, 229) | 89.5 (30, 224) | 89 (30, 211) | 85 (30, 202) | 119 (60, 258) | 120 (60, 282) | 90 (30, 240) |
| MAOIs | 65.5 (55, 76) | 65.5 (55, 76) | 108.5 (55, 349) | 65.5 (55, 76) | 65.5 (54, 166) | 68 (55, 275) | 66.5 (55, 148) |
| TRD approved medications§ | 30 (30, 60) | 30 (30, 60) | 30 (30, 60) | 30 (30, 60) | 30 (30, 60) | 30 (30, 60) | 30 (30, 60) |
| **Augmentation treatments*** |  |  |  |  |  |  |  |
| Anticonvulsants | 120 (47, 262) | 119 (43, 262) | 120 (53, 262) | 117.5 (37, 250) | 160 (79, 311) | 143 (60, 292) | 120 (57, 258) |
| Anxiolytics | 90 (30, 199.5) | 90 (30, 200) | 90 (30, 194.5) | 88 (30, 182) | 123 (60, 281) | 111 (58, 249) | 87 (30, 197) |
| Antipsychotics | 102 (51, 223) | 112 (53, 233) | 107.5 (53, 223) | 99.5 (48, 214) | 148 (79, 258) | 125.5 (60, 242) | 100 (51, 208) |
| Lithium | 65.5 (30, 234) | 80.5 (30, 258.5) | 72 (30, 173) | 60 (30, 205) | 114 (60, 262) | 111 (43, 215) | 79.5 (30, 196) |
| Thyroid hormone | 143 (34, 286) | 147 (34, 286) | 116.5 (56, 251) | 131.5 (30, 286) | 286 (151, 438) | 247 (90, 386) | 176 (79, 357) |

SSRIs: Selective serotonin reuptake inhibitors; NDRIs: Norepinephrine and dopamine reuptake inhibitors; SNRIs: Serotonin and norepinephrine reuptake inhibitors; MAOIs: monoamine oxidase inhibitors; TRD: Treatment resistant depression. CBT: Cognitive behavioral therapy; ECT: Electroconvulsive therapy; VNS: Vagus nerve stimulation; rTMS: Repetitive transcranial magnetic stimulation.

*Medications of different types of antidepressants are shown Appendix Table 4. Augmentation treatments are shown in Appendix Table 5.

†Duration: Total number of days supplied.

^§^Included esketamine and olanzapine in combination with fluoxetine.

eTable 12. Mean gap of treatments during the MDD episode for the TRD patients in each sensitivity analysis‡

|  | **Base analysis, N = 2,409, mean (SD)** | **Sensitivity analysis 1 N = 2,512, mean (SD)** | **Sensitivity analysis 2 N = 3,184, mean (SD)** | **Sensitivity analysis 3 N = 2,784, mean (SD)** | **Sensitivity analysis 4 N = 1,217, mean (SD)** | **Sensitivity analysis 5 N = 2,474, mean (SD)** | **Sensitivity analysis 6 N = 4,101, mean (SD)** |
| --- | --- | --- | --- | --- | --- | --- | --- |
| **Antidepressants*** |  |  |  |  |  |  |  |
| SSRIs | 116.2 (145.4) | 115.7 (144.8) | 115.7 (144.0) | 112.2 (142.8) | 107.8 (132.6) | 97.8 (124.7) | 122.6 (147.2) |
| NDRIs | 54.8 (96.6) | 55.2 (98.3) | 53.4 (96.7) | 53.3 (96.4) | 53.9 (88.7) | 55.2 (89.6) | 53.5 (97.7) |
| SNRIs | 57.0 (99.3) | 56.8 (98.7) | 57.2 (98.9) | 53.8 (96.1) | 56.3 (94.8) | 50.8 (85.7) | 58.4 (101.1) |
| Serotonin modulators | 68.7 (115.0) | 68.9 (114.1) | 71.9 (117.1) | 68.5 (116.1) | 73.2 (113.9) | 68.9 (111.6) | 66.0 (111.5) |
| Norepinephrine-serotonin modulators | 41.0 (92.5) | 40.5 (90.7) | 41.1 (92.5) | 35.4 (84.5) | 37.0 (71.0) | 35.0 (76.0) | 36.4 (84.0) |
| Tricyclics and tetracyclics | 47.8 (92.5) | 47.1 (92.1) | 48.4 (97.2) | 44.6 (90.6) | 55.8 (111.7) | 54.4 (101.7) | 54.3 (103.9) |
| MAOIs | 4.0 (5.7) | 4.0 (5.7) | 8.0 (16.0) | 4.0 (5.7) | 116.5 (227.7) | 77.5 (185.9) | 5.5 (4.9) |
| TRD approved medications§ | 45.1 (118.9) | 44.9 (118.6) | 45.1 (118.9) | 44.7 (119) | 52.3 (117.9) | 42.3 (100.4) | 44.9 (118.6) |
| **Augmentation treatments*** |  |  |  |  |  |  |  |
| Anticonvulsants | 38.1 (71.8) | 37.5 (70.5) | 38.4 (71.6) | 34.3 (64.2) | 37.7 (68.2) | 35.2 (67.3) | 41.2 (72.3) |
| Anxiolytics | 58.9 (104.8) | 59.7 (105.2) | 55.7 (101.3) | 55.4 (99.0) | 67.3 (108.3) | 64.5 (110.1) | 57.7 (103.0) |
| Antipsychotics | 51.7 (92.5) | 51.1 (91.3) | 52.8 (96.1) | 51.5 (94.5) | 56.0 (95.4) | 49.3 (89.1) | 49.3 (90.7) |
| Lithium | 47.5 (99.3) | 48.4 (93.1) | 39.2 (87.5) | 35.3 (70.3) | 47.2 (78.7) | 35.1 (57.2) | 39.2 (85.3) |
| Thyroid hormone | 37.6 (52.4) | 36.0 (51.7) | 33.1 (48.8) | 44.6 (61.6) | 57.3 (65.3) | 60.1 (80.5) | 34.1 (51.3) |

SSRIs: Selective serotonin reuptake inhibitors; NDRIs: Norepinephrine and dopamine reuptake inhibitors; SNRIs: Serotonin and norepinephrine reuptake inhibitors; MAOIs: monoamine oxidase inhibitors; TRD: Treatment resistant depression. CBT: Cognitive behavioral therapy; ECT: Electroconvulsive therapy; VNS: Vagus nerve stimulation; rTMS: Repetitive transcranial magnetic stimulation.

*Medications of different types of antidepressants are shown Appendix Table 4. Augmentation treatments are shown in Appendix Table 5.

‡Gap: The date of the last claim + days supplied of the last claim – the date of the first claim – total number of days supplied.

^§^Included esketamine and olanzapine in combination with fluoxetine.

eTable 13. Median gap of treatments during the MDD episode for the TRD patients in each sensitivity analysis‡

|  | **Base analysis, N = 2,409, median (IQR)** | **Sensitivity analysis 1 N = 2,512, median (IQR)** | **Sensitivity analysis 2 N = 3,184, median (IQR)** | **Sensitivity analysis 3 N = 2,784, median (IQR)** | **Sensitivity analysis 4 N = 1,217, median (IQR)** | **Sensitivity analysis 5 N = 2,474, median (IQR)** | **Sensitivity analysis 6 N = 4,101, median (IQR)** |
| --- | --- | --- | --- | --- | --- | --- | --- |
| **Antidepressants*** |  |  |  |  |  |  |  |
| SSRIs | 53 (7, 171) | 53 (7, 170) | 54 (7, 172) | 50 (6, 165) | 50 (10, 157) | 45 (8, 143) | 59.5 (8, 188) |
| NDRIs | 11 (0, 63) | 11 (0, 62) | 9 (0, 57) | 9 (0, 60) | 15 (0, 66) | 17 (0, 68) | 10 (0, 59) |
| SNRIs | 13 (0, 65) | 14 (0, 64) | 14 (0, 64) | 12 (0, 58) | 15 (1, 63) | 15 (1, 59) | 14 (0, 65) |
| Serotonin modulators | 10 (0, 88.5) | 11 (0, 89) | 12 (0, 94.5) | 10 (0, 88.5) | 13 (0, 101.5) | 15 (0, 93) | 9 (0, 83) |
| Norepinephrine-serotonin modulators | 1 (0, 35) | 2 (0, 35) | 1 (0, 32) | 0 (0, 27) | 7 (0, 44) | 4 (0, 29) | 0 (0, 29) |
| Tricyclics and tetracyclics | 4 (0, 50) | 3 (0, 48.5) | 3 (0, 48.5) | 2 (0, 46) | 5.5 (0, 39) | 9 (0, 61) | 4 (0, 59) |
| MAOIs | 4 (0, 8) | 4 (0, 8) | 0 (0, 8) | 4 (0, 8) | 4 (0, 233) | 0 (0, 8) | 6 (0, 10) |
| TRD approved medications^§^ | 0 (0, 8.5) | 0 (0, 11) | 0 (0, 8.5) | 0 (0, 8.5) | 0 (0, 25) | 0 (0, 25) | 0 (0, 6) |
| **Augmentation treatments*** |  |  |  |  |  |  |  |
| Anticonvulsants | 7 (0, 46) | 7 (0, 45.5) | 9 (0, 46) | 5.5 (0, 44) | 13.5 (0, 47.5) | 9.5 (0, 44) | 10 (0, 50) |
| Anxiolytics | 9 (0, 71) | 9 (0, 71.5) | 8 (0, 67.5) | 7 (0, 66) | 14 (0, 86) | 12 (0, 80) | 7 (0, 66) |
| Antipsychotics | 6 (0, 62) | 6 (0, 61) | 7 (0, 60) | 5.5 (0, 61) | 8.5 (0, 68) | 8 (0, 58) | 6 (0, 57) |
| Lithium | 4.5 (0, 42) | 5.5 (0, 59) | 4.5 (0, 39) | 0 (0, 39) | 8 (0, 66) | 5 (0, 48) | 5 (0, 40) |
| Thyroid hormone | 2 (0, 70) | 2 (0, 70) | 1.5 (0, 54) | 1 (0, 82) | 43 (0, 114) | 37 (0, 70) | 7 (0, 49) |

SSRIs: Selective serotonin reuptake inhibitors; NDRIs: Norepinephrine and dopamine reuptake inhibitors; SNRIs: Serotonin and norepinephrine reuptake inhibitors; MAOIs: monoamine oxidase inhibitors; TRD: Treatment resistant depression. CBT: Cognitive behavioral therapy; ECT: Electroconvulsive therapy; VNS: Vagus nerve stimulation; rTMS: Repetitive transcranial magnetic stimulation.

*Medications of different types of antidepressants are shown Appendix Table 4. Augmentation treatments are shown in Appendix Table 5.

‡Gap: The date of the last claim + days supplied of the last claim – the date of the first claim – total number of days supplied.

^§^Included esketamine and olanzapine in combination with fluoxetine.


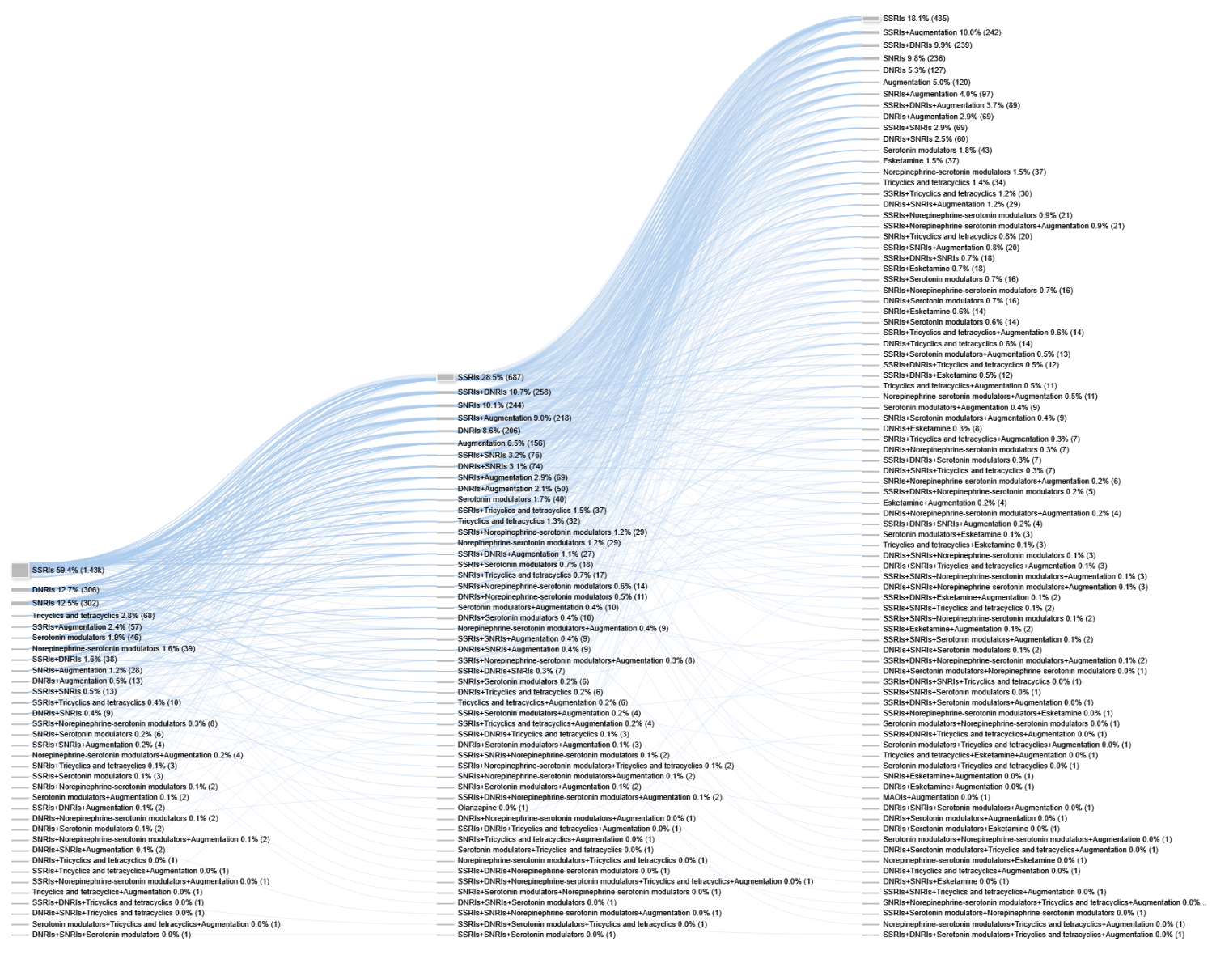


**Second-line to third-line**

Mean duration: 194.7 ± 151.9

Median duration: 142 (77, 279)

**Third-line**

**First-line to second-line**

Mean duration: 163.1 ± 135.9

Median duration: 113 (60, 224)

eFigure 1. Sankey plot for treatment trajectories of TRD patients (Overall, N = 2,409)


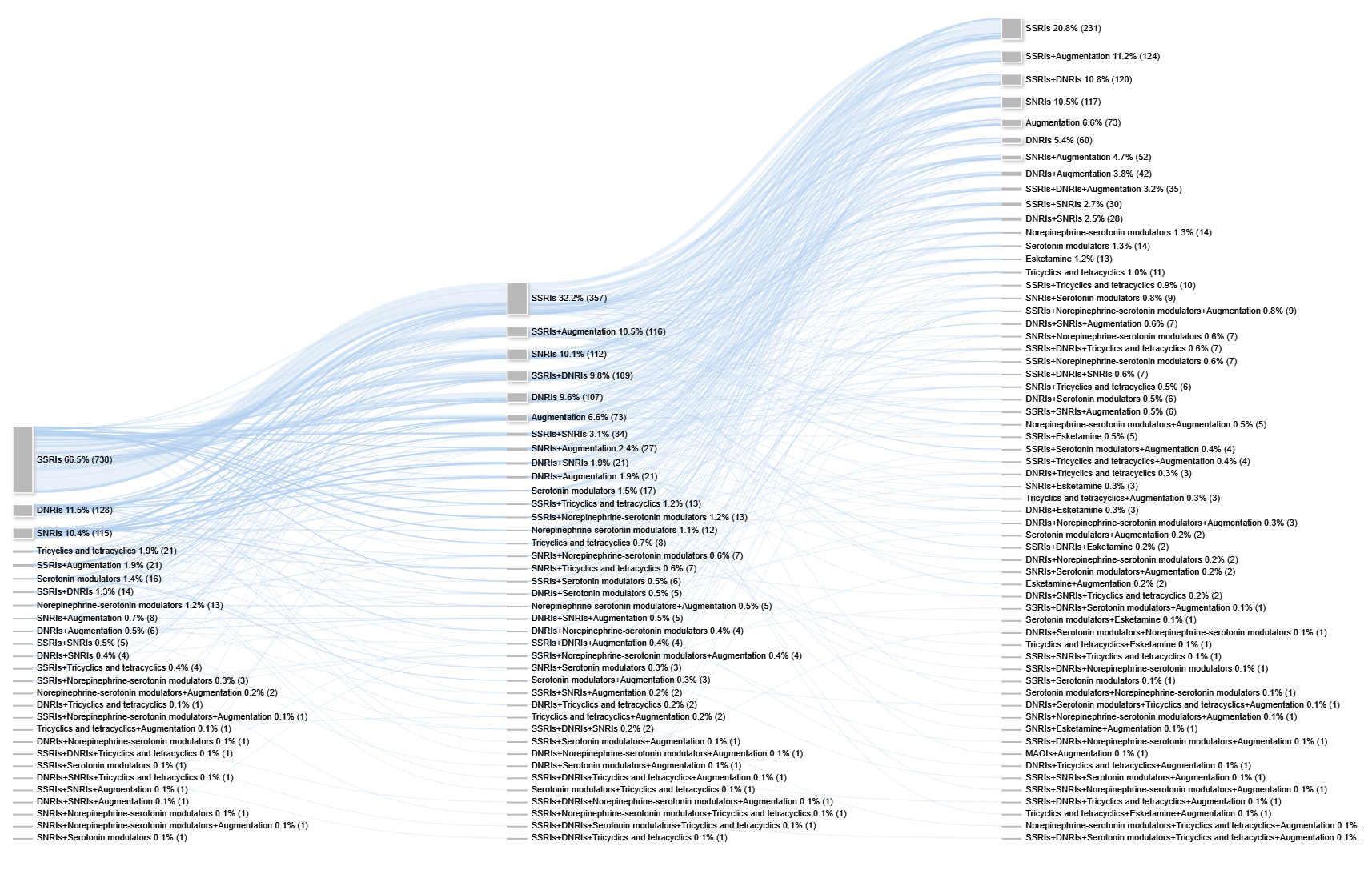


**Second-line**

**Third-line**

**First-line**

eFigure 2. Sankey Plot for treatment trajectories of TRD patients aged between 18 and 35 years (N = 1,110)

**Second-line**

**Third-line**

**First-line**


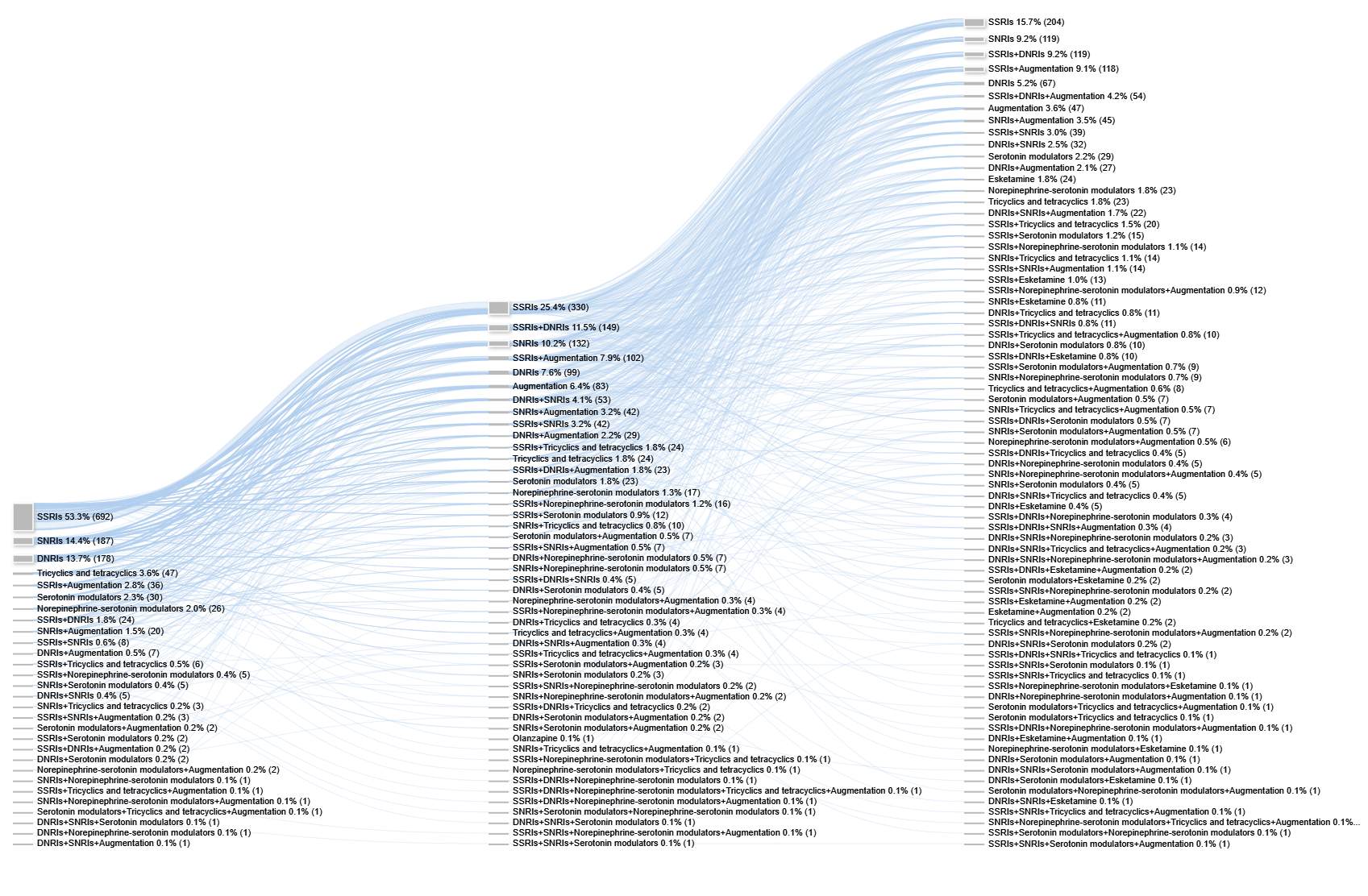


eFigure 3. Sankey Plot for treatment trajectories of TRD patients aged 35 years or older (N = 1,299)


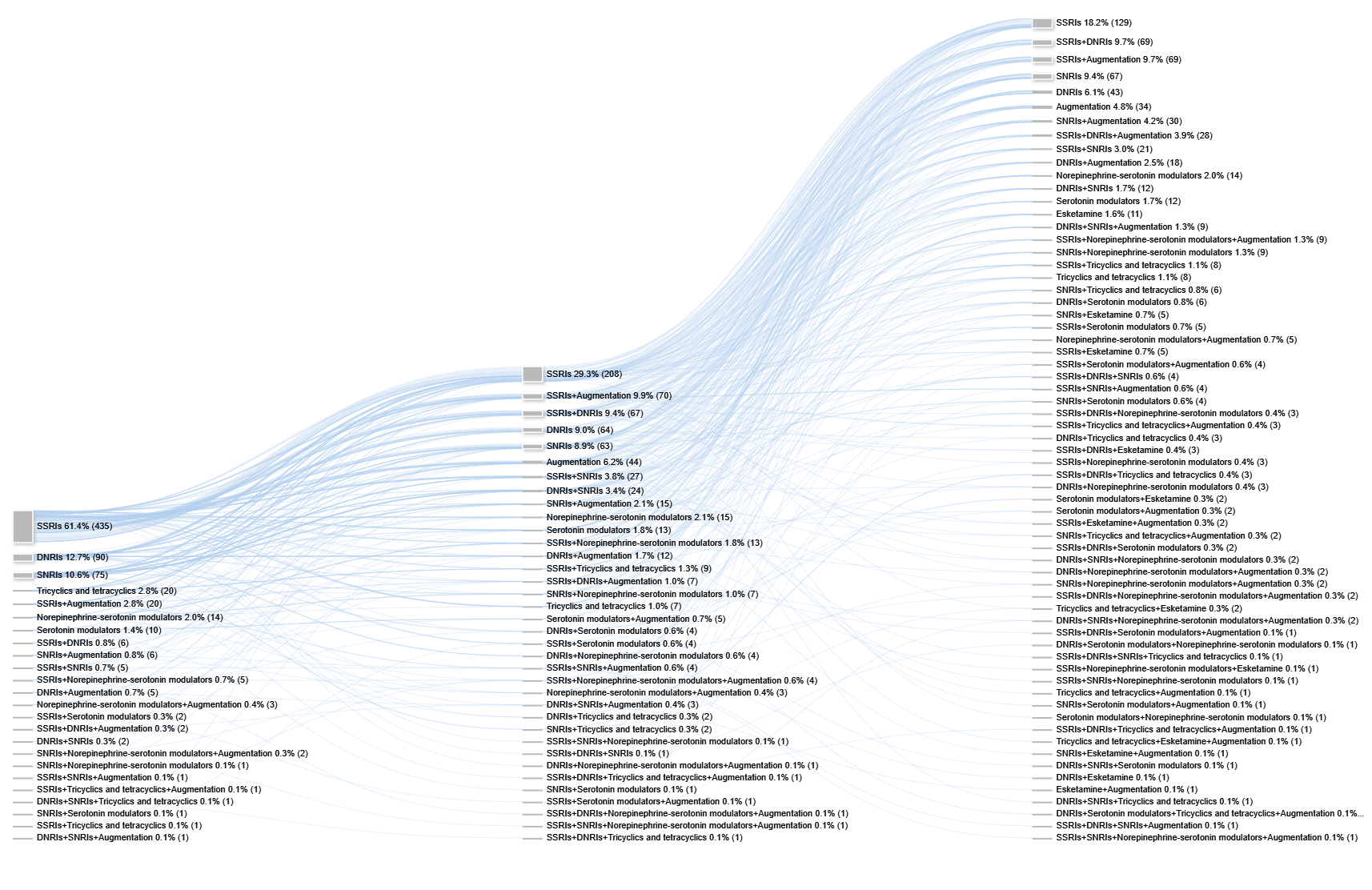


**Second-line**

**Third-line**

**First-line**

eFigure 4. Sankey Plot for treatment trajectories of male TRD patients (N = 709)

**Second-line**

**Third-line**

**First-line**


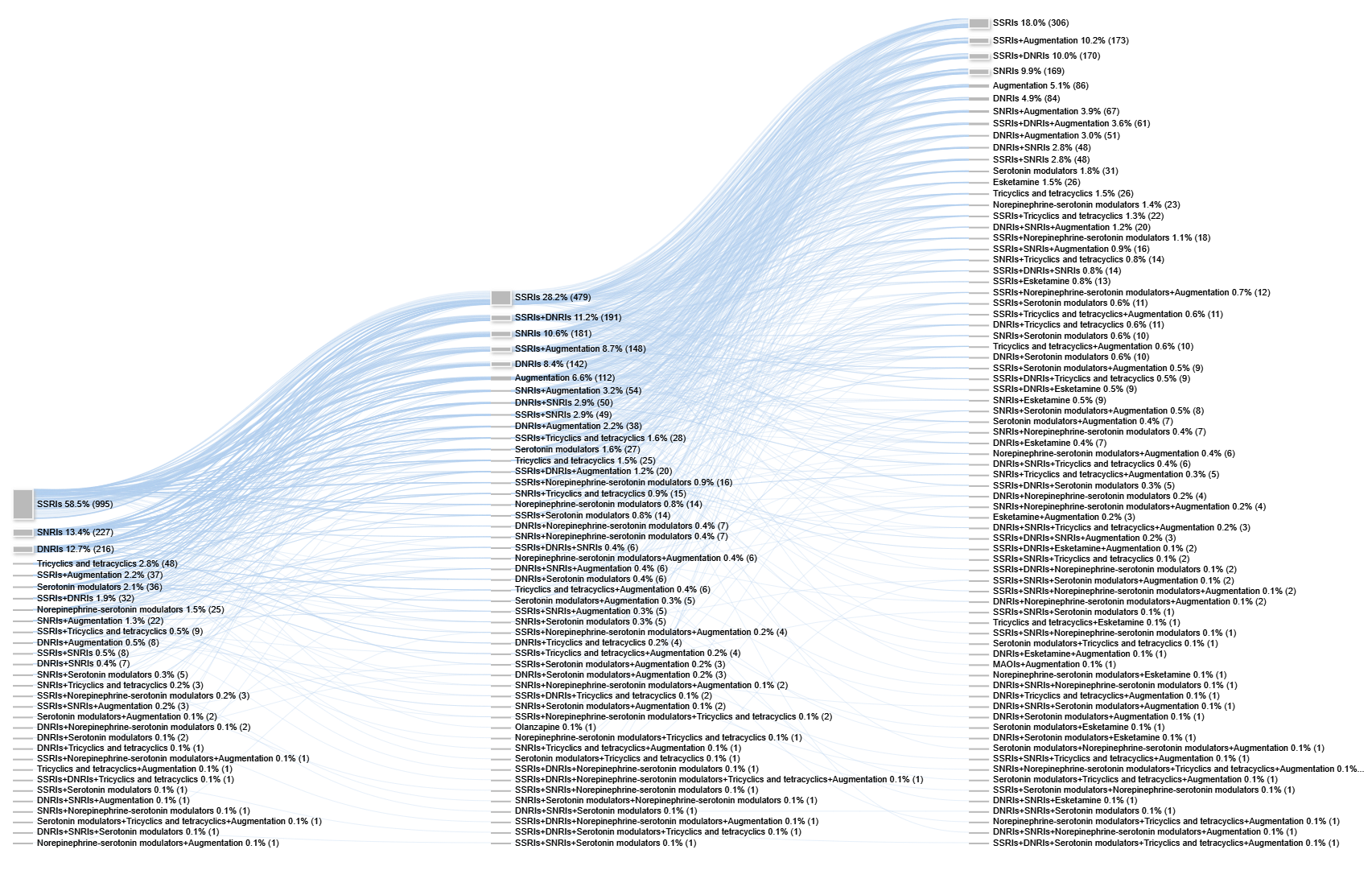


eFigure 5. Sankey Plot for treatment trajectories of female TRD patients (N = 1,700)


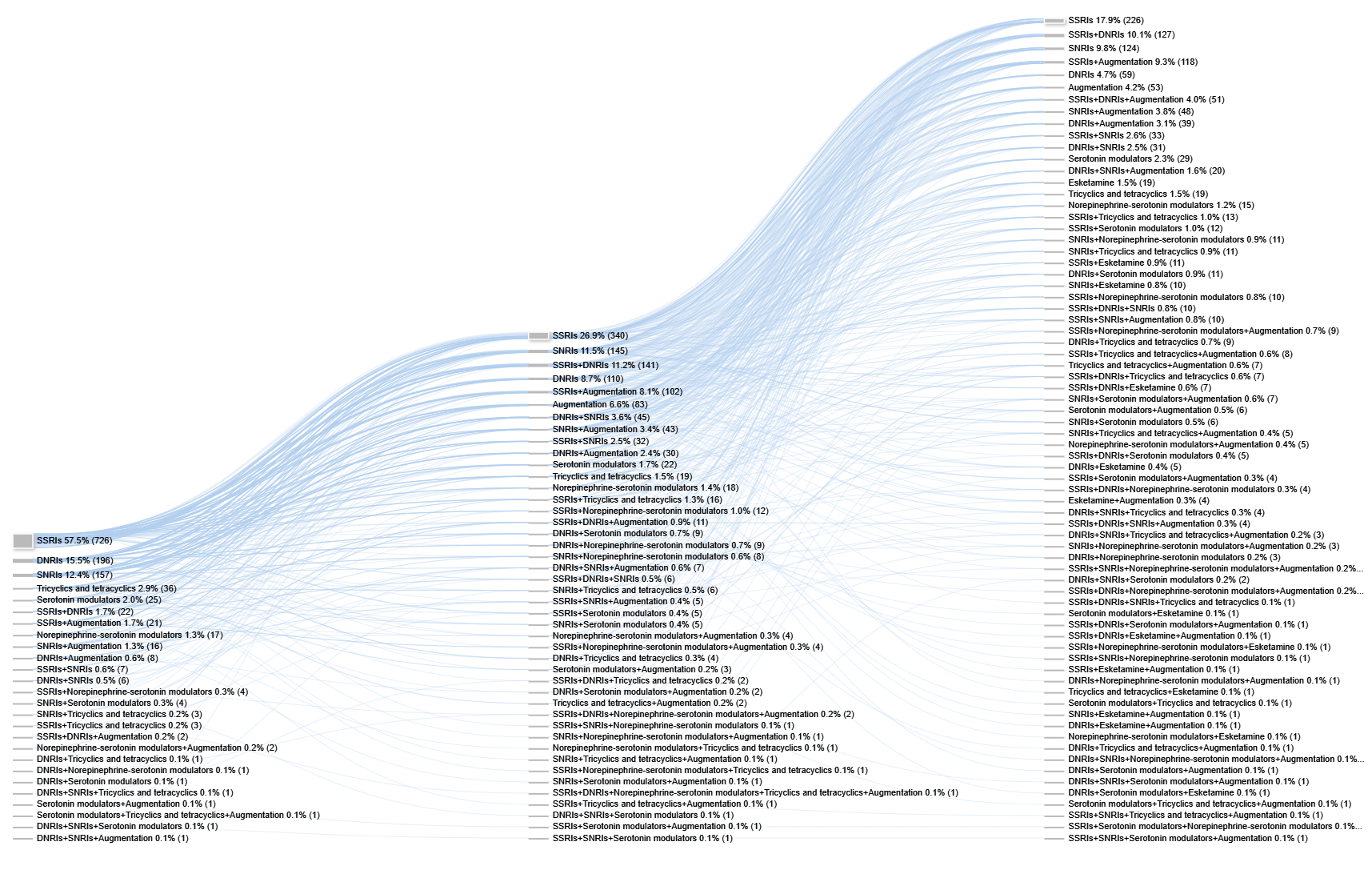


**Second-line**

**Third-line**

**First-line**

eFigure 6. Sankey Plot for treatment trajectories of TRD patients without anxiety (N = 1,263)

**Second-line**

**Third-line**

**First-line**


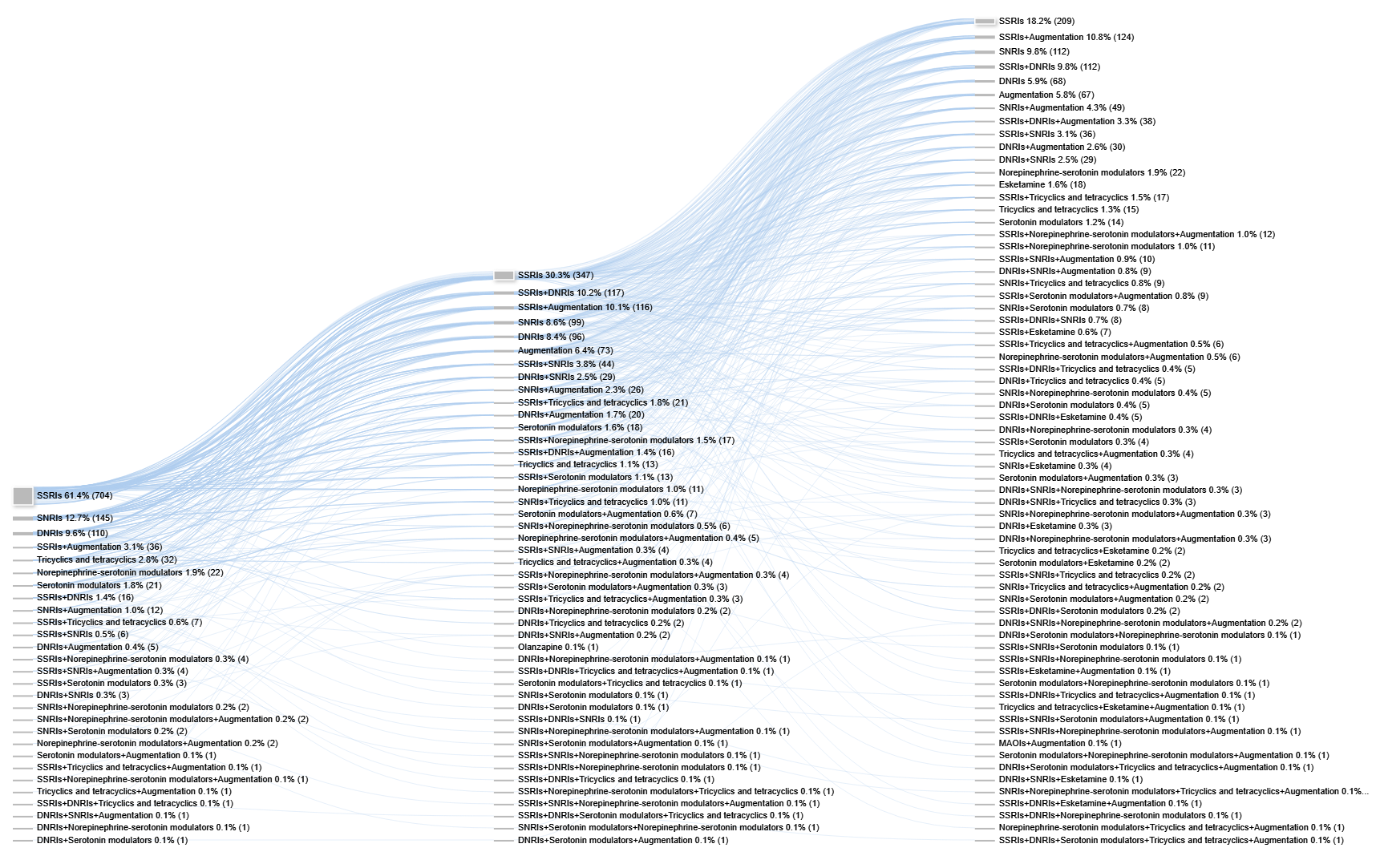


eFigure 7. Sankey Plot for treatment trajectories of TRD patients with anxiety (N = 1,146)
